# Rab32 GTPase, as a direct target of miR-30b/c, controls the intracellular survival of *Burkholderia pseudomallei* by regulating phagosome maturation

**DOI:** 10.1371/journal.ppat.1007879

**Published:** 2019-06-14

**Authors:** Zhi-qiang Hu, Cheng-long Rao, Meng-ling Tang, Yu zhang, Xiao-xue Lu, Jian-gao Chen, Chan Mao, Ling Deng, Qian Li, Xu-hu Mao

**Affiliations:** Department of Clinical Microbiology and Immunology, College of Pharmacy and Medical Laboratory & Southwest Hospital, Third Military Medical University (Army Medical University), Chongqing, China; INSERM U1220, FRANCE

## Abstract

*Burkholderia pseudomallei* is a gram-negative, facultative intracellular bacterium, which causes a disease known as melioidosis. Professional phagocytes represent a crucial first line of innate defense against invading pathogens. Uptake of pathogens by these cells involves the formation of a phagosome that matures by fusing with early and late endocytic vesicles, resulting in killing of ingested microbes. Host Rab GTPases are central regulators of vesicular trafficking following pathogen phagocytosis. However, it is unclear how Rab GTPases interact with *B*. *pseudomallei* to regulate the transport and maturation of bacterial-containing phagosomes. Here, we showed that the host Rab32 plays an important role in mediating antimicrobial activity by promoting phagosome maturation at an early phase of infection with *B*. *pseudomallei*. And we demonstrated that the expression level of Rab32 is increased through the downregulation of the synthesis of miR-30b/30c in *B*. *pseudomallei* infected macrophages. Subsequently, we showed that *B*. *pseudomallei* resides temporarily in Rab32-positive compartments with late endocytic features. And Rab32 enhances phagosome acidification and promotes the fusion of *B*. *pseudomallei*-containing phagosomes with lysosomes to activate cathepsin D, resulting in restricted intracellular growth of *B*. *pseudomallei*. Additionally, Rab32 mediates phagosome maturation depending on its guanosine triphosphate/guanosine diphosphate (GTP/GDP) binding state. Finally, we report the previously unrecognized role of miR-30b/30c in regulating *B*. *pseudomallei*-containing phagosome maturation by targeting Rab32 in macrophages. Altogether, we provide a novel insight into the host immune-regulated cellular pathway against *B*. *pseudomallei* infection is partially dependent on Rab32 trafficking pathway, which regulates phagosome maturation and enhances the killing of this bacterium in macrophages.

## Author summary

*Burkholderia pseudomallei* is a gram-negative intracellular bacterium and the etiological agent of melioidosis. Little is known about the host innate immune system, which is engaged in a continuous battle against this pathogen and may contribute to the outcomes of melioidosis. Recently, Rab32, a Rab GTPase was shown to be a critical regulator of a host defense pathway against intracellular bacterial pathogens. However, the exact mechanism of how Rab32 contributes to the restriction of intracellular pathogens is not completely understood. In this study, we determined that the infection of macrophages with *B*. *pseudomallei* resulted in the upregulation of Rab32 expression through the inhibition of miR-30b/30c expression. Subsequently, Rab32 is recruited to the *B*. *pseudomallei*-containing phagosomes and promotes the fusion of the phagosomes with lysosomes, which results in the increased exposure of *B*. *pseudomallei* to lysosomal acid hydrolases CTSD, thus limiting the intracellular growth of *B*. *pseudomallei* at an early phase of infection in macrophages. Our findings establish for the first time that Rab32 plays an important role in suppressing the intracellular replication of *B*. *pseudomallei* by modulating phagosome maturation in macrophages, providing a new insight into the host defense mechanisms against *B*. *pseudomallei* infection.

## Introduction

Host innate immune cells, particularly professional phagocytes, possess a wide range of antimicrobial defense mechanisms to eliminate the invading microbes [1]. Phagocytosis, an evolutionarily conserved process of the innate immune response, plays an indispensable role in the host-defense responses against a wide range of intracellular pathogens [2]. After the phagocytosis of pathogens by macrophages, the resulting intracellular vacuoles are termed as phagosomes [3]. The phagosomes are then processed in a series of interactions with different endosomes, resulting in the progressive acidification of the phagosome lumen and activation of the hydrolytic enzymes, which finally leads to their acquisition of degradative and antimicrobial properties [4]. Rab GTPases, well known as central regulators of vesicular trafficking, are closely linked to the endocytosis and trafficking of intracellular pathogens [5, 6]. To date, many studies have shown that the Rab GTPases are involved in the formation and maturation of phagosomes. Phagosome purification combined with proteomic analysis have identified several Rab GTPases that associate with phagosomes. Of these, Rab5 and Rab7 are the most well-characterized Rab proteins with regard to their localization to phagosomes and their role in phagosome maturation [3]. Rab5 is associated with early phagosomes and facilitates the recruitment of its effector proteins, early endosome associated antigen (EEA1) [7]. During the period of maturation, the phagosomes lose Rab5 and acquire Rab7, which allows phagosomes to interact with the late endosomes and lysosomes [8]. Although there are over 70 Rab GTPases identified in mammalian cells and more than 20 on phagosomal membranes, but only a few of them have been investigated with regard to their function in phagosome maturation.

*Burkholderia pseudomallei* is a facultative intracellular pathogen that causes the fatal infectious disease melioidosis, which has broad-spectrum clinical manifestations including pneumonia, localized abscesses, and septicemia [9]. Melioidosis is highly endemic across tropical and subtropical regions, especially in Southeast Asia and northern Australia. The common routes of infection with environmental *B*. *pseudomallei* are cutaneous inoculation, ingestion, and inhalation [10, 11]. *B*. *pseudomallei* can invade and survive in both phagocytic and non-phagocytic cells [12, 13]. Some mechanisms that allow phagocytes to limit the growth of *B*. *pseudomallei* have been elucidated [14–18]. Moreover, many studies have shown that *B*. *pseudomallei* can escape from the phagosome into the cytosol of phagocytic cells where it replicates and acquires actin-mediated motility, avoiding killing by the autophagy-dependent process [19–22]. However, the exact mechanistic details of *B*. *pseudomallei* adapt to the intraphagosomal environment and manipulate the phagocytic process remains unknown. Therefore, to identify host cell molecules and pathways utilized by *B*. *pseudomallei* for intracellular survival, we initially investigated the localization and expression of 19 Rab GTPases, which are critical regulators of membrane trafficking pathways. Using overexpression of EGFP-tagged Rab GTPases, we observed considerable localization of Rab32 with *B*. *pseudomallei*-containing phagosomes, and increased Rab32 expression.

Rab32 is a multifunctional protein, depending upon its cellular localization and the cell type. It is well established that Rab32 is involved in the biogenesis of lysosome-related organelles (LROs) such as melanosomes, T cells, and platelet-dense granules [23, 24]. In addition, Rab32 is also implicated in host response to bacterial infection. Hoffmann et al. previously showed, Rab32 associates with *Legionella*-containing vacuoles and appear to promote the intracellular growth of *L*. *pneumophila* [25]. Some recent studies have demonstrated that Rab32 is recruited to the vacuoles containing bacterial pathogens, such as *S*. *Typhi* and *L*. *monocytogenes* [26, 27], and it is essential to protect host cells from these pathogens. However, the exact mechanism of how Rab32 contributes to the restriction of intracellular pathogens is not completely understood.

In addition to Rab GTPases can regulate phagosome maturation, increasing evidence indicates that microRNAs (miRNAs) are not only crucial regulators involved in modulating host innate immune responses to pathogens [28–30], but also play critical roles in regulating the phagolysosomal pathway. However, the specific role of miRNAs in the regulation of membrane trafficking during *B*. *pseudomallei* infection is largely unknown. In this study, we aimed to explore the role of Rab32 in host-dependent immune mechanisms against *B*. *pseudomallei* infection. We found that *B*. *pseudomallei* upregulates the expression of Rab32 in infected macrophages by downregulating the expression of miR-30b/30c. Moreover, Rab32 is a functional GTPase that is required for limiting intracellular replication of *B*. *pseudomallei* by promoting the fusion of phagosomes with lysosomes.

## Results

### Rab32 expression is upregulated and recruited to the *B*. *pseudomallei*-containing phagosomes during macrophage infections

**Fig 1:**
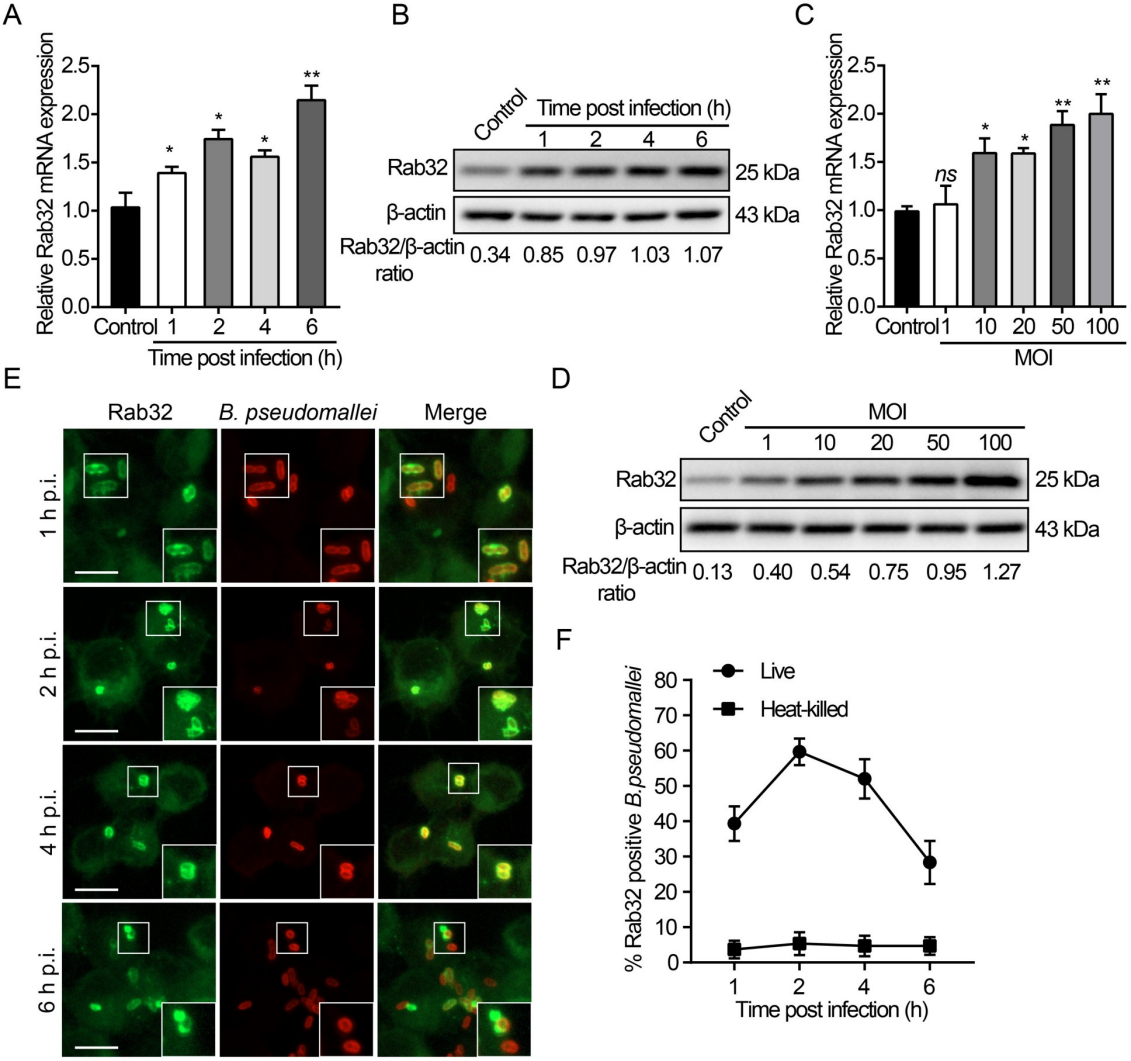
*B*. *pseudomallei* infection increased the expression of Rab32 and its recruitment into *B*. *pseudomallei*-containing phagosomes. (A-D) *B*. *pseudomallei* infection increased the expression levels (mRNA and protein) of Rab32 in RAW264.7 cells. RAW264.7 cells were treated with *B*. *pseudomallei* (MOI = 10:1) for 0, 1, 2, 4, and 6 h, or at MOI = 0, 1, 10, 20, 50, and 100 for 2 h. (E) RAW264.7 cells were infected with *B*. *pseudomallei* (MOI = 10:1) and imaged at the indicated time points: 1 to 6 h and stained with an anti-Rab32 antibody (green) anti-*B*. *pseudomallei* antibody (red). Images show maximum-intensity projections of confocal Z-stacks. Scale bar is 5 μm. (F) The percentage of association of live and heat-killed *B*. *pseudomallei* phagosomes with Rab32 at each time point is depicted in this panel. Scale bar is 5 μm. *ns*, no significant difference. Data are representative of at least three independent experiments (*P<0.05, **P<0.01).

Previous studies indicate that Rab GTPases have the ability to target microbial pathogen-containing vacuoles and coordinate a host-mediated defense response to control intracellular pathogen replication [26, 27, 31–33]. To investigate whether Rab32 is involved in preventing *B*. *pseudomallei* from replicating within the host cells, we used RAW 264.7 macrophages infected with *B*. *pseudomallei* and measured the expression of Rab32 by using quantitative real-time-PCR (qRT-PCR) and western blot analyses. As shown in Fig 1A and 1B, there was a gradual increase over time in the ratio of Rab32 to β-actin in *B*. *pseudomallei* infection (multiplicity of infection, MOI = 10:1) as compared with an uninfected control in RAW264.7 cells. Consistent with the observed Rab32 which is upregulated in time course experiments; similar results were observed when MOI dependency was tested at 2 h post-infection (Fig 1C and 1D).

As host Rab GTPases regulate intracellular vesicular trafficking through specific interactions with vesicle membranes, we examined the localization of Rab32 in cultured macrophages infected with *B*. *pseudomallei* for different periods of time. We found that enhanced green fluorescent protein (EGFP)-Rab32 was strongly recruited to the *B*. *pseudomallei*-containing phagosomes 1 h to 6 h post-infection (p.i.) i.e., after bacterial internalization (Fig 1E). Maximum recruitment was at 2 h p.i. (60.4 ± 4.2%) and recruitment gradually decreased after 4 h of infection (Fig 1F). Interestingly, we found that Rab32 recruitment was specific for live *B*. *pseudomallei* as a heat-killed *B*. *pseudomallei* was unable to efficiently recruit Rab32 to phagosomes (S1A Fig), compared to live *B*. *pseudomallei* only 5.3 ± 2.1% of heat-killed *B*. *pseudomallei* colocalized with Rab32 in time course experiments (Fig 1F). In addition, we also found that heat-killed *B*. *pseudomallei* had no effect on the regulation of Rab32 mRNA and protein expression (S1B and S1C Fig). Furthermore, heat-killed *B*. *pseudomallei* showed the same internalization rate as the live bacterium (S1D and S1E Fig). Collectively, these results indicate that viable *B*. *pseudomallei* can be specifically sensed by the macrophages to trigger Rab32 expression and recruitment into the *B*. *pseudomallei*-containing phagosomes.

### MiR-30b and miR-30c expression is downregulated after infection of macrophages by *B*. *pseudomallei*

**Fig 2:**
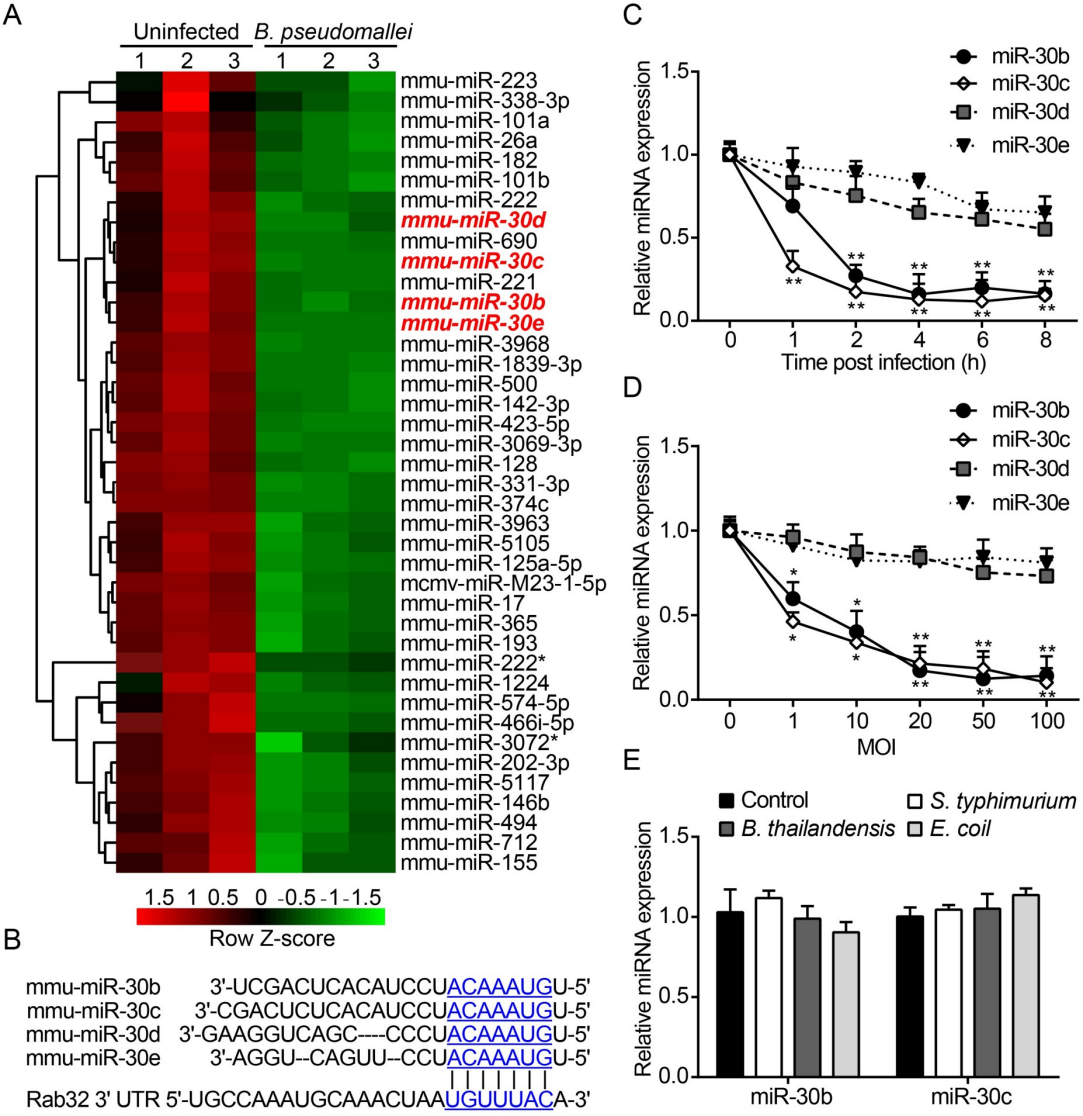
MiR-30b and miR-30c are downregulated after *B*. *pseudomallei* infection. (A) Total RNA from RAW264.7 cells were infected with *B*. *pseudomallei* for 4 h to perform microarray assay. Hierarchical clustering analysis was performed to show downregulated miRNAs by *B*. *pseudomallei* infection. (B) The region of the mouse Rab32 mRNA 3′UTR predicted to be targeted by miR-30b, miR-30c, miR-30d, and miR-30e, respectively (TargetScan 6.2). (C and D) Confirmation of microarray results by qRT-PCR. qRT-PCR analysis of the expression levels of miR-30b, miR-30c, miR-30d, and miR-30e in RAW264.7 cells infected with *B*. *pseudomallei* (MOI = 10) for 0, 1, 2, 4, 6, and 8 h, or at MOI = 0, 1, 10, 20, 50, and 100 for 4 h. (E) Expression of miR-30b and miR-30c in RAW264.7 cells infected with *B*. *thailandensis*, *S*. *typhimurium* and *E*. *coli* (MOI = 10). *P<0.05, **P<0.01. Experiments performed in triplicates showed consistent results.

To investigate the underlying mechanisms of *B*. *pseudomallei* infection which upregulates the expression of Rab32, we used miRNA microarrays to analyze genome-wide miRNA expression profiles in RAW264.7 cells were infected with *B*. *pseudomallei* at an MOI of 10. As shown in S1 Table, among the 124 differentially expressed miRNAs, 3 miRNAs were significantly upregulated (*P* < 0.05), while 121 miRNAs were significantly downregulated (*P* < 0.05). Fig 1A shows the heat map of top 40 miRNAs that were significant downregulated in response to *B*. *pseudomallei* infection at 4 h. MiRNAs negatively regulate the expression of target genes mainly by interaction in their 3' untranslated region (UTR). Thus, we screened for miRNAs whose expression downregulated after *B*. *pseudomallei* infection by using target prediction tools: TargetScan and miRDB, as candidate miRNAs for the increased Rab32 expression specific. We found members of the miR-30 family were predicted to target the 3' UTR of Rab32 mRNA (Fig 2B).

The miR-30 family is an important member of miRNA family, which contains five members (miR-30a, miR-30b, miR-30c, miR-30d, and miR-30e) and located at different genomic positions [34]. To confirm the validity of our microarray data, the expression levels of key members of the mi-30 family of miRNAs (miR-30b/30c/30d/30e) were examined by qRT-PCR in macrophages after *B*. *pseudomallei* infection. As shown in Fig 2C, the expression of miR-30b/30c rapidly decreased in response to *B*. *pseudomallei* infection as early as 1 h p.i., but miR-30d/30e did not decrease significantly. Additionally, similar results were obtained when *B*. *pseudomallei* infected RAW264.7 cells at 2 h p.i., which showed a gradual decrease in the expression of miR-30b/30c in an MOI-dependent manner (Fig 2D). We then also examined whether there were differential responses to the expression levels of miR-30 family members between live and heat-killed *B*. *pseudomallei*. Interestingly, the qRT-PCR analysis indicated that stimulation of RAW264.7 cells with heat-killed *B*. *pseudomallei* were unable to downregulate the expression of these miRNAs (S2A and S2B Fig). The observed responses are consistent with results showing that heat-killed *B*. *pseudomallei* were unable to induce Rab32 expression and recruitment into the bacteria-containing phagosomes (S1A and S1C Fig). Moreover, in order to examined whether the downregulation of miR-30b/30c was specific to *B*. *pseudomallei*, we used *Burkholderia thailandensis* (an avirulent bacterium closely related to *B*. *pseudomallei*) and other Gram-negative bacteria, like *Salmonella typhimurium* and *Escherichia coli* as controls. We found no significant changes in the expression levels of miR-30b/30c after infection with *S*. *typhimurium*, *B*. *thailandensis*, and *E*. *coli* (Fig 2E). Taken together, these results show that the expression levels of miR-30b/30c decrease in response to *B*. *pseudomallei* infection.

### Rab32 is a novel target of miR-30b and miR-30c

**Fig 3:**
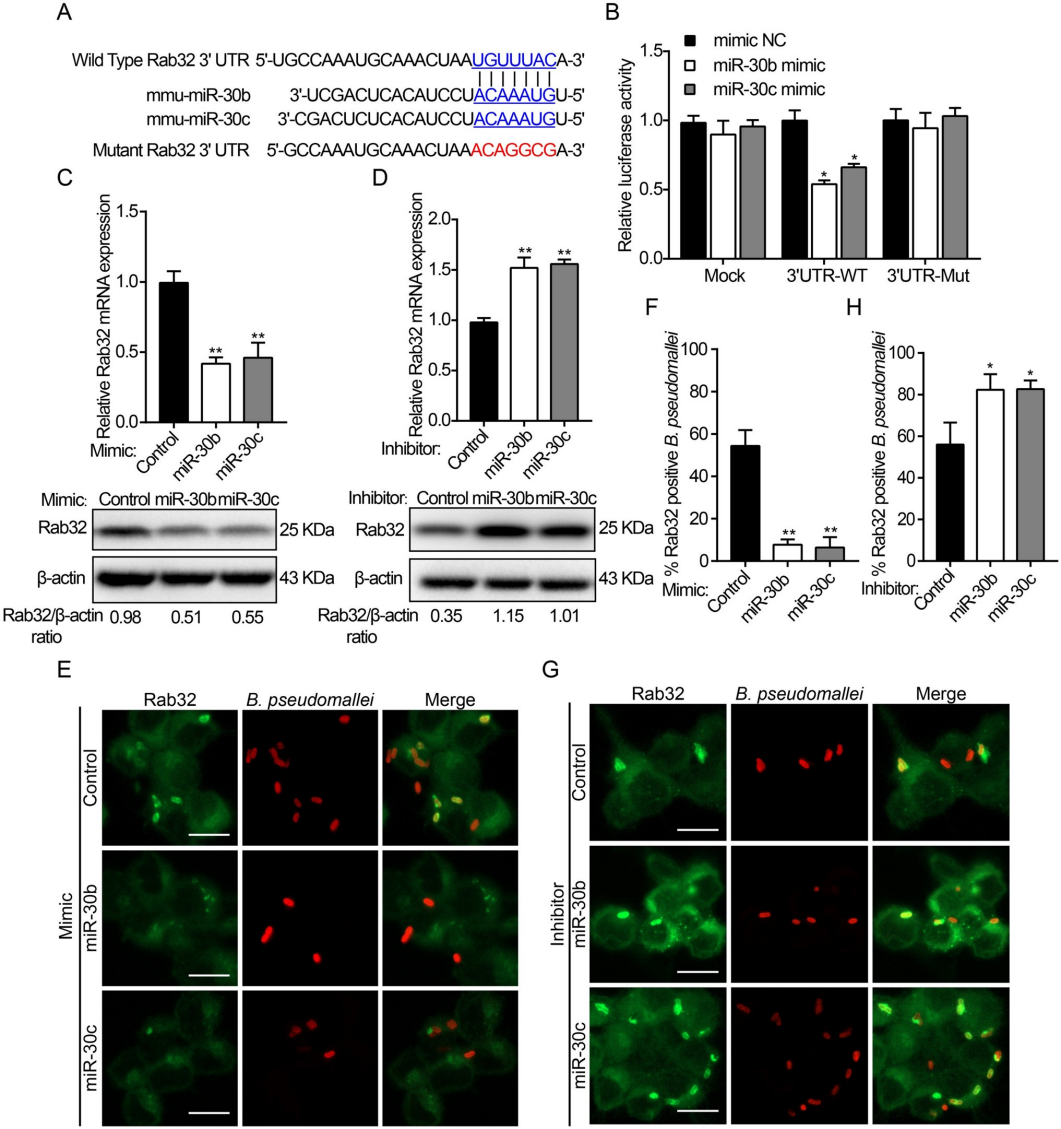
Rab32 is a direct target of miR-30b and miR-30c. (A) Sequences of miR-30b and miR-30c, and the potential binding site at the 3′UTR of Rab32. Also shown are nucleotides mutated in Rab32-3′UTR mutant. (B) HEK293 cells were co-transfected with luciferase reporter vectors carrying Rab32 WT or mutant constructs (Mut), along with an miR30b or miR-30c mimic or control. Luciferase activity was normalized to the activity of Renilla luciferase. (C and D) RAW264.7 cells were transiently transfected with miR-30b and miR-30c mimic, inhibitor, or control for 24 h. The mRNA and protein levels of Rab32 were determined by qRT-PCR and Western blot, respectively. (E) RAW264.7 cells transfected with miR-30b/30c mimic and control were infected with *B*. *pseudomallei* for 2 h, then fixed, and stained with an anti-Rab32 (green) or anti-*B*. *pseudomallei* antibody (red). (F) Quantitative analysis of Rab32 associated with *B*. *pseudomallei* phagosomes. (G) RAW264.7 cells transfected with miR-30b/30c inhibitor and control were infected with *B*. *pseudomallei* for 2 h, and association of Rab32 to *B*. *pseudomallei* phagosomes as in E. (H) Quantitative analysis of Rab32 associated with *B*. *pseudomallei* phagosomes as indicated in F. Scale bar is 5 μm. Data are representative of at least three independent experiments (*P<0.05, **P<0.01).

MiRNAs are small non-coding RNAs that negatively regulate post-transcriptional expression of target genes, which guide the binding of the miRNA-induced silencing complex (miRISC) to regions of partial complementarity located mainly within 3' untranslated region (3'UTR) of target mRNAs, resulting in mRNA degradation and/or translational repression [35, 36]. To identify whether Rab32 could be regulated by miR-30b and miR-30c. Firstly, we generated luciferase reporter vectors (pmirGLO) containing the wild-type or mutant 3′UTR of Rab32 mRNA predicted seed sequence (Fig 3A). HEK293 cells were co-transfected with control or miR-30b/30c mimics and luciferase reporter vectors containing either wild-type or mutant sequence of Rab32 3′UTR. Luciferase activity was effectively suppressed in the cells co-transfected with miR-30b/30c mimics and the wild-type reporter vectors, while no obvious changes in luciferase activity were observed in the control or mutant reporter vector groups (Fig 3B). The above data indicated that miR-30b/30c could bind to the 3′UTR predicted site of Rab32, resulting in the suppression of Rab32 expression levels. Next, we explored whether overexpression of miR-30b/30c could decrease Rab32 expression in RAW264.7 cells. We found that mRNA and protein levels of Rab32 strongly decreased when treated with miR-30b/30c mimics as compared to the miRNA control (Fig 3C and S3A Fig). By contrast, Rab32 mRNA levels and protein levels were enhanced when the cells were transfected with miR-30b/30c inhibitors (Fig 3D and S3B Fig). These results clearly demonstrate that Rab32 is specific targets of miR-30b/30c in RAW264.7 cells.

Since our results showed that *B*. *pseudomallei* infection upregulates Rab32 expression and recruits Rab32 to the bacterium-containing vacuole (Fig 1B and 1E). Thus, we investigated whether miR-30b/30c affects the colocalization of Rab32 and *B*. *pseudomallei* phagosomes in RAW264.7 cells. As shown in Fig 3E and 3F by confocal microscopy, in RAW264.7 cells after transfection with the miR-30b/30c mimics, about 7% of *B*. *pseudomallei* was in Rab32-positive phagosomes at 2 h after infection, whereas transfection with the miR-30b/30c inhibitors significantly increased the proportion of Rab32-positive *B*. *pseudomallei* (approximately 83%; Fig 3G and 3H). Collectively, these results demonstrated that miR-30b/30c suppressed the expression of Rab32 through mRNA degradation, thus affecting the recruitment of Rab32 to *B*. *pseudomallei*-containing phagosomes.

### *B*. *pseudomallei* resides in the Rab32-coated compartment with late endosomal features

**Fig 4:**
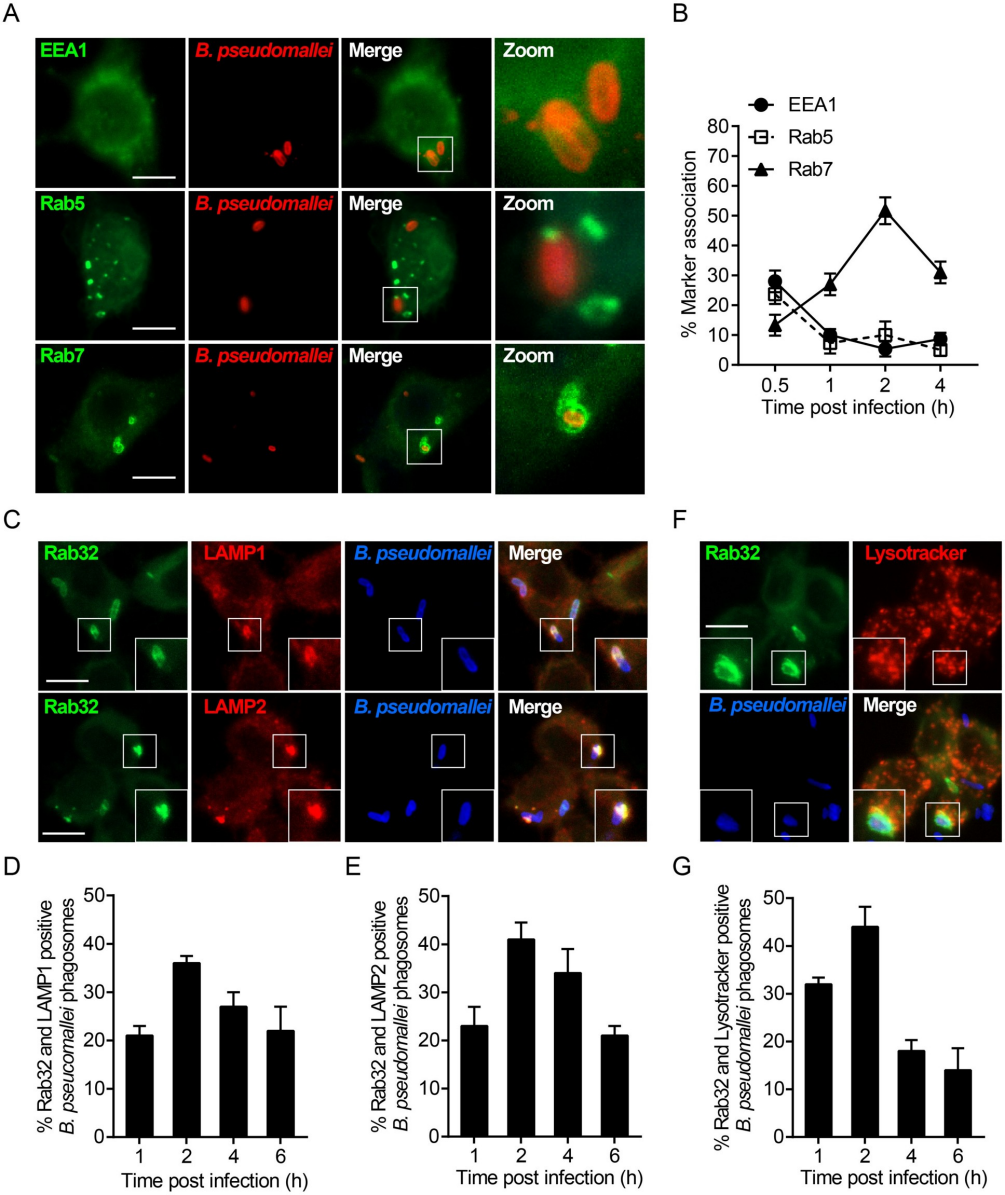
*B*. *pseudomallei* resides in a Rab32-labeled compartment with late endosomal features. (A) RAW264.7 cells were infected with *B*. *pseudomallei*, at an MOI of 10 for 2 h. Cells were stained with anti-EEA1, anti-Rab5, and anti-Rab7 antibodies (green), or anti-*B*. *pseudomallei* antibody (red) and colocalization was determined by confocal microscopy. Scale bar is 5 μm. (B) The percentage of EEA1, Rab5, or Rab7 colocalizing with *B*. *pseudomallei* phagosomes was enumerated by fluorescence microscopy. At least 100 bacterial phagosomes were counted for each time point. Results are represented as mean ± S.D. of three independent experiments. (C) RAW264.7 cells expressing EGFP-Rab32 were infected with *B*. *pseudomallei* for 2 h, afterwards cells were subjected to immunofluorescence for LAMP1 or LAMP2 (red) and stained with an anti-*B*. *pseudomallei* antibody (blue). (D) RAW264.7 cells expressing EGFP-Rab32 were infected with *B*. *pseudomallei*, fixed, and immunostained for LAMP1 (red) and *B*. *pseudomallei* (blue). *B*. *pseudomallei* phagosomes that colocalized with Rab32 and LAMP1 were quantified. Results are the means ± SD of three independent experiments. (E) Quantitative analysis of *B*. *pseudomallei* phagosomes that colocalized with Rab32 and LAMP2 as indicated in D. (F) RAW264.7 cells expressing EGFP-Rab32 were incubated with 50 nM Lysotracker (red) for 1 h before infection with *B*. *pseudomallei* for 2 h. Cells were stained with anti-*B*. *pseudomallei* antibody (blue) and colocalization was determined by confocal microscopy. (G) Quantitative analysis of *B*. *pseudomallei* phagosomes that colocalized with Rab32 and Lysotracker as indicated in D. Symbols represent single bacteria or distinct *B*. *pseudomallei* groups. Scale bar is 5 μm.

Shortly after infection, Rab32 was recruited to the *B*. *pseudomallei*–containing phagosomes and remained associated with these phagosomes for several hours after infection (Fig 1C and 1D). To monitor the trafficking stages of the bacteria–containing phagosomes in the endocytic pathway, we next investigated the features of the Rab32-positive compartments in *B*. *pseudomallei* infection. The association of markers for early (EEA1, Rab5) and late (Rab7) endosomes with *B*. *pseudomallei* phagosomes were assessed from 0.5 to 4 h after infection (S4A–S4C Fig). We found that EEA1 association with *B*. *pseudomallei* phagosomes was transient and confocal microscopy revealed detectible levels of EEA1 on phagosomal membranes at very early time-points (Fig 4A and S4A Fig). Consistent with these observations, Rab5 association, was transient and occurred only during the first 30 min (23.6 ± 0.8%) after infection with *B*. *pseudomallei* (Fig 4B and S4B Fig). Conversely, late endosomal marker Rab7 associated with *B*. *pseudomallei* phagosomes from 30 min, peaked at around 2 h (51.6 ± 2.4%) and declined afterward (Fig 4B and S4C Fig). Therefore, the presence of Rab7 on *B*. *pseudomallei* phagosomes suggested that fusion of late endosomes with the *B*. *pseudomallei* phagosomes can occur at 2 h after infection.

To further define the stage of the phagocytic sequence of Rab32-positive phagosomes, we next examined the time-course of Rab32 and other late endosomal markers (LAMP1, LAMP2) colocalizations with *B*. *pseudomallei* phagosomes (S4D and S4E Fig). The percentage of Rab32-positive phagosomes colocalization with LAMP1 and LAMP2 was assessed via quantitative analysis. At 1 h p.i., only small amounts of Rab32-positive phagosomes were found to associate with LAMP1 and LAMP2 (21.1 ± 1.8%, 23.4 ± 3.6%, respectively), indicating that fusion with late endosomes was minimal at this time. However, by 2 h p.i. the majority of Rab32-positive phagosomes had accumulated LAMP1 and LAMP2 (36.6 ± 1.2%, 41.2 ± 3.1%, respectively), indicating late endosome fusion had taken place (Fig 4D and 4E). Moreover, we also evaluated the acidification of Rab32-positive phagosomes by using the acidotropic probe Lysotracker (S4F Fig). We observed that 44.2 ± 4.3% of Rab32-positive phagosomes colocalized with lysotracker at 2 h p.i., but thereafter this percentage decreases (Fig 4F and 4G). Taken together, these results indicated that a substantial fraction of Rab32-positive phagosomes harboring *B*. *pseudomallei* appears to fuse with acidic late endosomes at early stages of infection.

### Rab32 is crucial for limiting the intracellular survival of *B*. *pseudomallei*

**Fig 5:**
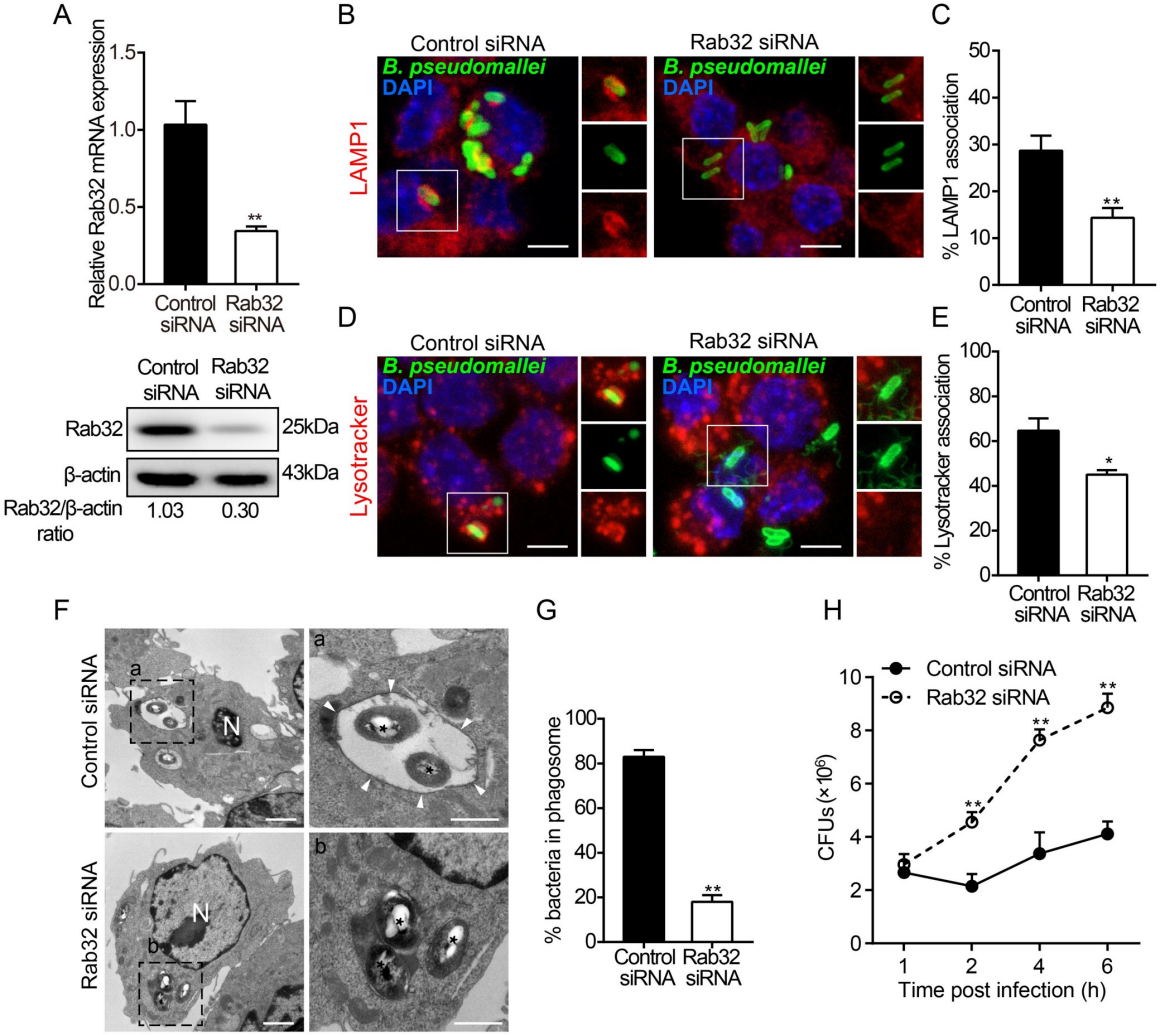
Rab32-positive phagosomes were recruited to limit the growth of *B*. *pseudomallei*. (A) The relative mRNA or protein concentrations of Rab32 were measured in RAW264.7 cells after transfection with 100 nM Rab32 specific siRNA or control RNA for 24 h. (B and C) Representative images of control and Rab32 silenced cells were infected with *B*. *pseudomallei* for 2 h, and stained with anti-LAMP1 antibody (red), or anti-*B*. *pseudomallei* antibody (green) or DAPI (blue). Quantitative analysis of LAMP1 fluorescence intensity associated with *B*. *pseudomallei* phagosomes. Scale bar is 5 μm. (D and E) Association of LAMP2 with *B*. *pseudomallei* as in B and a quantitative analysis of LAMP2 association with *B*. *pseudomallei* phagosomes as in C. Scale bar is 5 μm. (F) Representative TEM images of control and Rab32 silenced RAW264.7 cells infected with *B*. *pseudomallei* (asterisks) for 2 h showing differences in the intracellular location of *B*. *pseudomallei*. (white arrowheads = phagosome membrane; N = nucleus). Scale bar is 5 μm. (G) Quantitative analysis of bacteria associated with single-membrane phagosomes. At least 150 bacteria were quantified. (H) Intracellular *B*. *pseudomallei* burdens in Rab32 silenced and control RAW264.7 cells at the indicated times after infection. Data is shown as the mean ± SD of three independent experiments. *P<0.05, **P<0.01.

To further understand the functional consequences of Rab32 in *B*. *pseudomallei* infection, we investigated the effects of knockdown of Rab32 expression on *B*. *pseudomallei* phagosomes. We used a small interfering RNA (siRNA)-mediated knockdown of Rab32 expression in RAW264.7 macrophages. To test the silencing efficiency, the expression levels of Rab32 were analyzed by qRT-PCR and Western blot assays. We found that the mRNA (3-fold, *p* = 0.002) and protein (3.3-fold, *p* = 0.005) levels of endogenous Rab32 were significantly decreased in RAW264.7 cells transfected with Rab32 siRNA (Fig 5A). Next, we examined whether Rab32 is required for *B*. *pseudomallei* phagosomes maturation. As shown in Fig 5B and 5C, Rab32 knockdown significantly reduced the percentage of association between *B*. *pseudomallei* phagosomes and LAMP1 compared to control siRNA at 2 h p.i. (2-fold, *p* = 0.003). Similarly, the percentage of LysoTracker-positive *B*. *pseudomallei* phagosomes were also obviously lower in Rab32 siRNA transfected macrophages than that in control siRNA transfected macrophages (1.4-fold, *p* = 0.02; Fig 5D and 5E). These results suggested that Rab32 may interfere with the maturation of *B*. *pseudomallei* phagosomes. Additionally, using transmission electron microscopy (TEM), we further observed the transport of *B*. *pseudomallei* phagosomes in Rab32-depleted RAW264.7 cells. At 2 h p.i., TEM results showed that about 80% of the *B*. *pseudomallei* were intact and surrounded by the single-membrane phagosomes in control siRNA-transfected macrophages (Fig 5F and 5G). Conversely, in the Rab32 siRNA transfected macrophages, most bacteria had escaped from the phagosomes into the cytoplasm at 2 h p.i. (4.6-fold, *p* = 0.008; Fig 5F and 5G). In agreement with the TEM observed results, colony forming unit (CFU) assays showed that Rab32 silencing resulted in significantly increased intracellular growth of *B*. *pseudomallei* compared to control siRNA treated macrophages (2 fold at 2 h, *p* = 0.007 and 2.2 fold at 6 h, *p* = 0.008; Fig 5H). And the growth of *B*. *pseudomallei* was not caused by differences in internalization, because the Rab32 knockdown did not affect the extent of internalization (S5A Fig). Thus, our data indicated that Rab32 is not only involved in *B*. *pseudomallei*-containing phagosomes formation, but is also indispensable for controlling intracellular replication of *B*. *pseudomallei* in macrophages.

### Rab32 facilitates the fusion of *B*. *pseudomallei*-containing phagosomes with lysosomes

**Fig 6:**
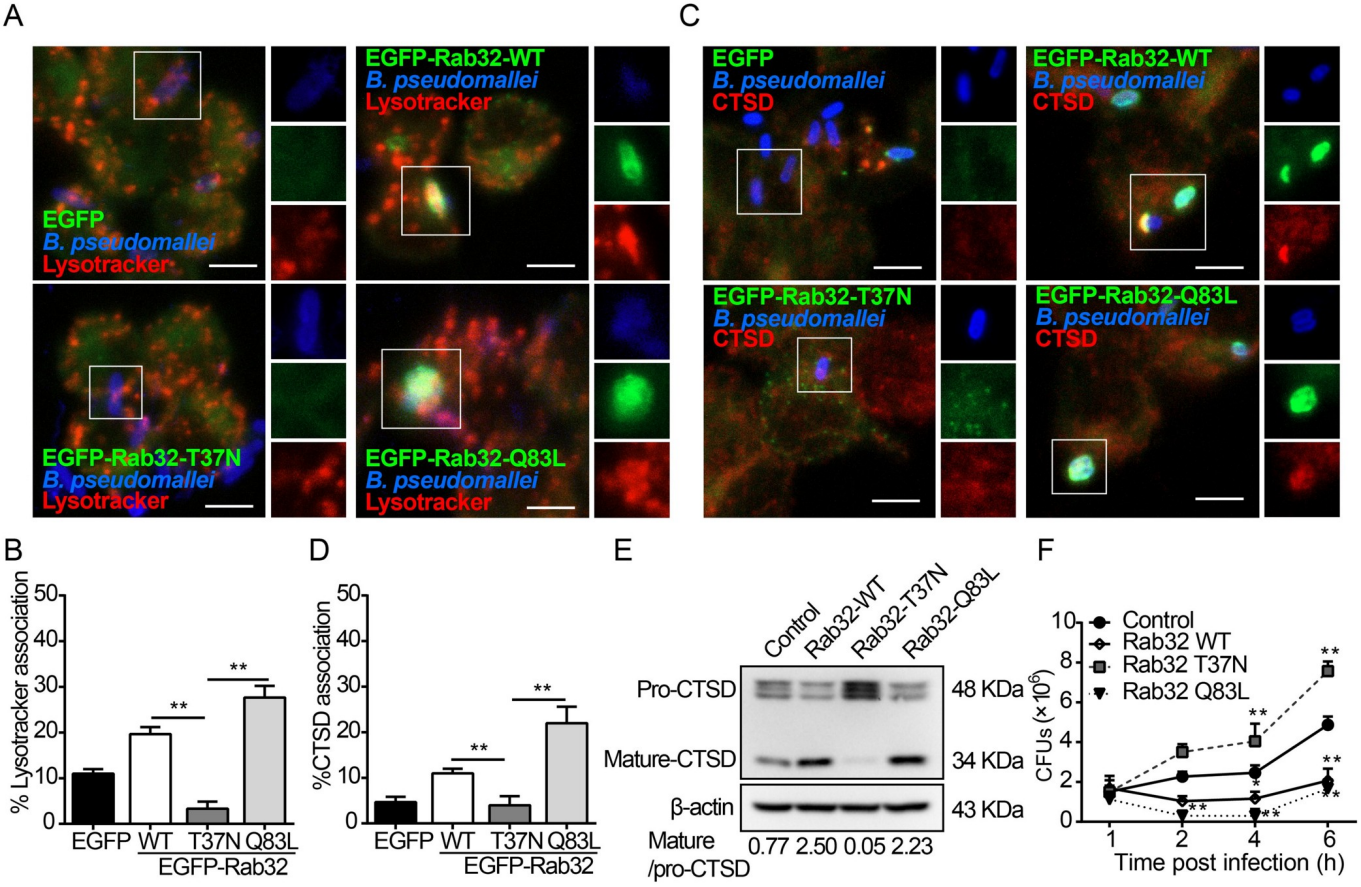
Rab32 increases fusion of *B*. *pseudomallei*-containing phagosomes with lysosomes. (A) RAW264.7 cells expressing the different constructs were incubated with 50 nM Lysotracker for 1 h before infection with *B*. *pseudomallei*, and stained for *B*. *pseudomallei* (blue). Scale bar is 5 μm. (B) Quantitative analysis of *B*. *pseudomallei* phagosomes associated with Lysotracker. Results are represented as mean ± S.D. of three independent experiments. (C) RAW264.7 cells transfected with the indicated constructs were infected with *B*. *pseudomallei*, and cells were fixed and stained for cathepsin D (CTSD, red) and *B*. *pseudomallei* (blue). Scale bar is 5 μm. (D) Analysis of the number of *B*. *pseudomallei* phagosomes positive for CTSD. Results are represented as mean ± S.D. of three independent experiments. (E) Rab32 and its mutants were overexpressed in RAW264.7 cells and cells were infected with *B*. *pseudomallei* (MOI = 10) for 2 h, total cellular extracts were prepared and subjected to western blotting using antibody against CTSD. (F) RAW264.7 cells selected for the expression of EGFP, EGFP-Rab32, EGFP-Rab32-T37N, and EGFP-Rab32-Q83L were infected with *B*. *pseudomallei* at the indicated time points, cells were lysed, and CFU determined. Results are represented as mean ± SD for at least three separate experiments (*P<0.05, **P<0.01).

Considering that the majority of the Rab32-positive phagosomes harboring *B*. *pseudomallei* had late endosomal features and Rab32 contributes to restricting the intracellular growth of *B*. *pseudomallei* in macrophages. Therefore, we hypothesized that Rab32 is involved in the fusion of *B*. *pseudomallei*-containing phagosomes with lysosomal compartment for more efficient degradation of invading *B*. *pseudomallei*. Rab GTPases cycle between the GDP- and GTP-bound states and are regulated by guanine nucleotide exchange factors (GEFs) and GTPase activating proteins (GAPs) that influence their subcellular localization and functions [5]. Therefore, we explore the impacts of overexpression of EGFP-Rab32 and mutants on phagosome maturation. Expression of the EGFP tagged Rab32-T37N (inactive GDP-bound mutant), Rab32-Q83L (active GTP-bound mutant) and wild-type Rab32 caused no obvious effect on the internalization of *B*. *pseudomallei* in RAW264.7 cells (S5B Fig). We then investigated whether the recruitment of Rab32 to the *B*. *pseudomallei* phagosome depends on its GTP/GDP binding state. Our results showed that overexpression of EGFP-Rab32-WT (4 fold, *p* = 0.0005) and EGFP-Rab32-Q83L (4.5 fold, *p* = 0.0002) significantly increase the recruitment of Rab32 to *B*. *pseudomallei*-containing phagosomes respectively, but not EGFP-Rab32-T37N (S6A and S6B Fig). In the process of phagosome maturation, acidification of the phagosome is required for efficient phago-lysosomal fusion. Indeed, we observed that the overexpression of EGFP-Rab32-WT (5.7 fold, *p* = 0.001) or EGFP-Rab32-Q83L (8.2 fold, *p* = 0.006) showed significant enhancement in the association between *B*. *pseudomallei* phagosomes and the acidotropic probe Lysotracker as compared to the EGFP-Rab32-T37N groups, respectively (Fig 6A and 6B). Moreover, the later stages of phagosome maturation are also characterized by the acquisition of lysosomal acid hydrolases, such as cathepsin D (CTSD, the lysosomal marker) [32]. Thus, we next examined the recruitment of CTSD to the *B*. *pseudomallei* phagosomes in RAW264.7 cells transfected with pEGFP-Rab32 and its mutant vectors. Expression of the dominant active mutant of EGFP-Rab32-Q83L significantly increased the association of endogenous CTSD with *B*. *pseudomallei* phagosomes (5.5 fold, *p* = 0.0006; Fig 6C and 6D). In contrast, the forced expression of the inactive mutant Rab32-T37N inhibited the recruitment of CTSD to phagosomes, compared to the EGFP-Rab32-WT groups (2.8 fold, *p* = 0.004; Fig 6C and 6D). The lysosomal enzyme CTSD is first synthesized as an inactive precursor (pro-CTSD), which is then cleaved to produce the mature active form of CTSD in acidic lysosomes [37].

In addition, the activation of CTSD is crucial for the effective elimination of intracellular pathogens in phagolysosomes. Hence, the intracellular processing status of CTSD could be further used as an indicator of maturation of *B*. *pseudomallei* phagosomes. Consistent with Lysotracker and CTSD localization, we found that the overexpression of EGFP-Rab32 and EGFP-Rab32-Q83L significantly increased the levels of mature/activated form of CTSD (Fig 6E), correlating with the increased killing of *B*. *pseudomallei* (Fig 6F). Conversely, overexpression of the EGFP-Rab32-T37N significantly blocks CTSD maturation (Fig 6E), leading to an increase in the number of intracellular *B*. *pseudomallei* (Fig 6F). These data indicate that Rab32 GTPase activity is involved in the fusion of *B*. *pseudomallei*-containing phagosomes with the degradative lysosomal compartment.

### MiR-30b and miR-30c regulate phagosome maturation against *B*. *pseudomallei* by targeting Rab32

**Fig 7:**
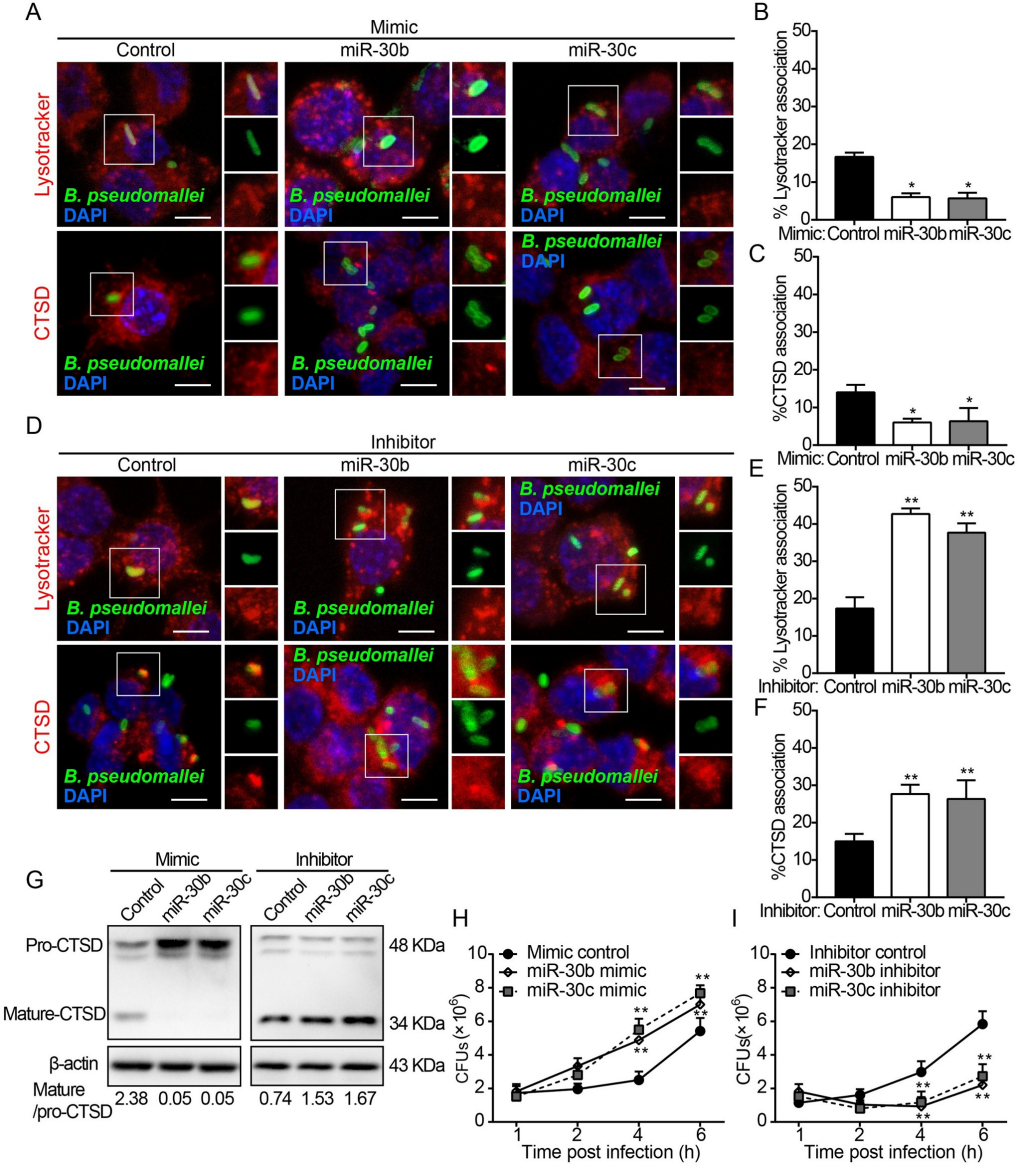
MiR-30b and miR-30c affect phagosome maturation and modulate *B*. *pseudomallei* intracellular survival in macrophages. (A) BMDMs transfected with control, miR-30b mimic, and miR-30c mimic were incubated with 50 nM Lysotracker (red) for 1 h before infection with *B*. *pseudomallei* for 2 h and fixed, stained with an anti-CTSD (red) or anti-*B*. *pseudomallei* (green). Scale bar is 5 μm. (B and C) Percent of *B*. *pseudomallei* phagosomes that colocalized with Lysotracker and CTSD at 2 h post-infection as in A. Results represented are of three independent experiments. (D) BMDMs were transfected by control, miR-30b inhibitor or miR-30c inhibitor for 24 h, followed by *B*. *pseudomallei* infection for 2 h, and were labeled with Lysotracker (red) or stained with an anti-CTSD (red). Scale bar is 5 μm. (E and F) Quantification showing the percentage of association of the bacteria phagosomes with Lysotracker and CTSD as in D. (G) BMDMs were transfected with control, miR-30b mimic, miR-30c mimic, miR-30b inhibitor, and miR-30c inhibitor for 24 h and then infected with *B*. *pseudomallei*. The expression of CTSD was determined by Western blot analysis. (H) BMDMs were transfected with control, miR-30b mimic, and miR-30c mimic at 100 nM for 24 h, and were then infected with *B*. *pseudomallei* for different periods of time (0, 1, 2, 4, and 6 h). Intracellular bacterial counts were determined. (I) After BMDMs were transfected with control, miR-30b inhibitor or miR-30c inhibitor for 24 h, intracellular survival of *B*. *pseudomallei* was detected as in H. Data are representative of at least three independent experiments (*P<0.05, **P<0.01).

Several reports have pointed to a role of mi-30 family members in the immune response to pathogens [38–41]. It therefore appeared likely that the differential regulation of mi-30b/30c influences the immune response to *B*. *pseudomallei* infection. Rab32 is a functional target of miR-30b/30c and is required for phagosome maturation. Next, we extended our analysis to primary bone marrow–derived macrophages (BMDMs) and examined whether miR-30b/30c also regulate the phagosome maturation in *B*. *pseudomallei* infection. Firstly, we further established the specificity of miR-30b/30c via overexpression of miR-30b/30c mimics or inhibitors in BMDMs. Consistent with previous observations, qRT-PCR and western blot analysis demonstrated that transfection of BMDMs with miR-30b/30c mimics decreased Rab32 mRNA and protein expression (S7A Fig), whereas transfection of BMDMs with miR-30b/30c inhibitors increased Rab32 mRNA and protein expression (S7B Fig). Similarly, we next tested miR-30b/30c effects on the colocalization of Lysotracker and CTSD with *B*. *pseudomallei*-containing phagosomes in BMDMs by immunofluorescence analysis. As shown in Fig 7A–7C, at 2 h p.i., transfection with miR-30b/30c mimics decreased the colocalization of *B*. *pseudomallei* phagosomes with Lysotracker (2.7-fold, *p* = 0.03; 2.9-fold, *p* = 0.02, respectively) and CTSD (2.3-fold, *p* = 0.02; 2.2-fold, *p* = 0.04, respectively) in BMDMs, when compared to the miR control. Conversely, the colocalization of Lysotracker (2.5 fold, *p* = 0.0002; 2.2 fold, *p* = 0.0007, respectively) and CTSD (1.9 fold, *p* = 0.0005; 1.8 fold, *p* = 0.0004, respectively) was markedly increased in *B*. *pseudomallei*-infected BMDMs transfected with miR-30b/30c inhibitors (Fig 7D–7F).

Additionally, since hydrolytic enzyme CTSD matures to the active form and obtains optimal antimicrobial activity in acidified lysosomes, we further investigated whether miR-30b/30c could affect the processing of CTSD in *B*. *pseudomallei*-infected BMDMs. Western blotting revealed that transfection with miR-30b/30c mimics significantly decreased the levels of mature/activated form of CTSD in BMDMs, whereas transfection with miR-30b/30c inhibitors had an inverse effect (Fig 7G). Furthermore, as shown in Fig 7H and 7I, compared with the miR control group, transfection of BMDMs with miR-30b/30c mimics raised the intracellular growth of *B*. *pseudomallei* (1.9 fold at 4 h, *p* = 0.004; 2.2 fold at 4 h, *p* = 0.002, respectively; Fig 7H), whereas transfection with miR-30b/30c inhibitors inhibited this growth (3.9-fold at 4 h, *p* = 0.005; 3.5-fold at 4 h, *p* = 0.008, respectively; Fig 7I), as assessed by the CFU assay. These data collectively suggested that miR-30b/30c negatively regulate the maturation of *B*. *pseudomallei*-containing phagosomes and intracellular antimicrobial activity, which was consistent with our previous data, and further supports that Rab32 is a direct target of miR-30b/30c.

## Discussion

**Fig 8:**
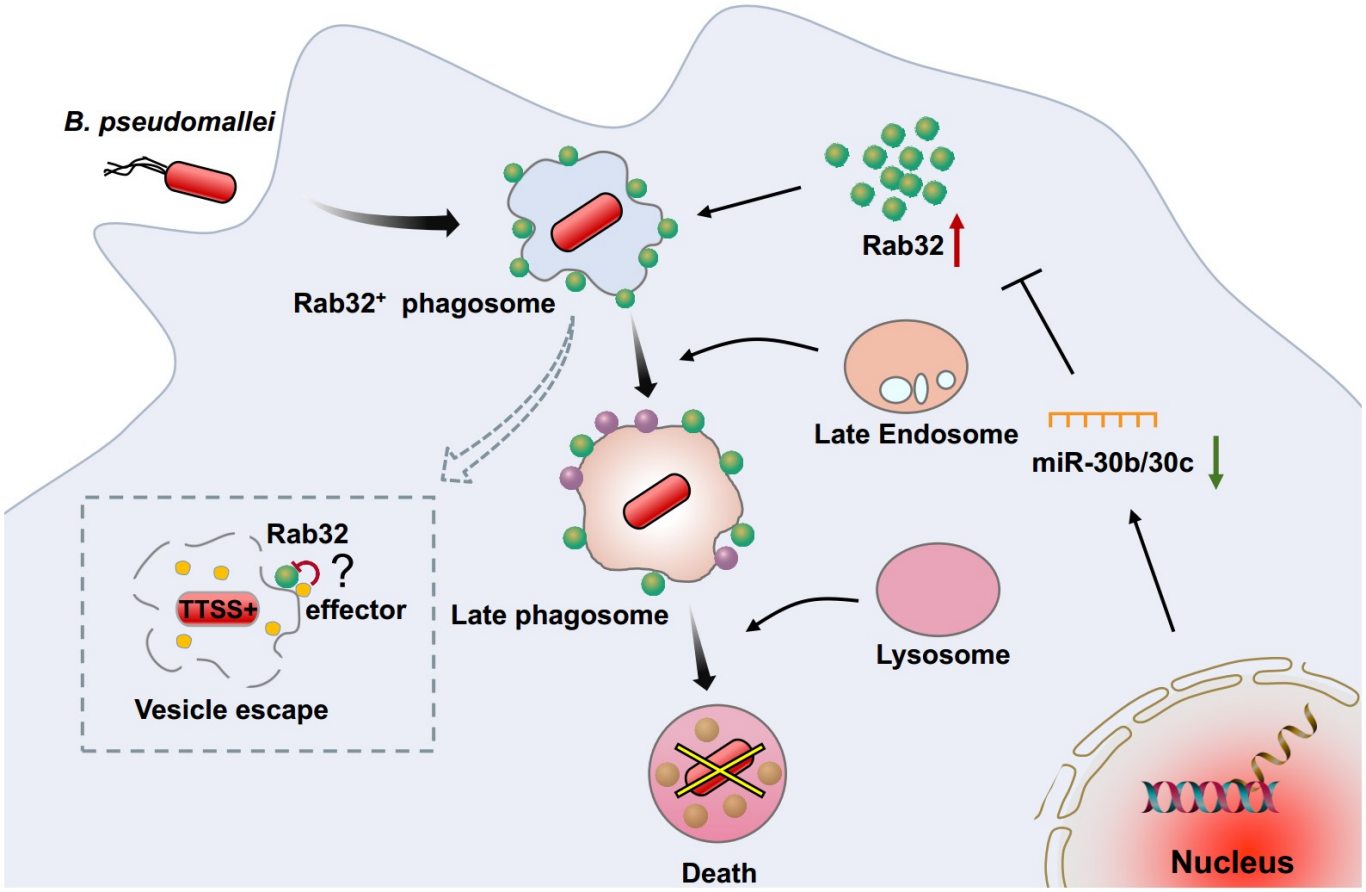
Schematic representation of Rab32 in the control of *Burkholderia pseudomallei* intracellular growth by macrophages. After internalization into macrophages, host cell responds to *B*. *pseudomallei* infection by downregulating the expression of miR-30b/30c, which results in an increase in Rab32 expression. Subsequently, Rab32 is recruited to the *B*. *pseudomallei*-containing phagosomes and promotes the transport to late endocytic compartment. Finally, Rab32 accelerates the *B*. *pseudomalle*i-containing phagosomes trafficking to the lysosomal compartment with degradative activity. Additionally, a portion of *B*. *pseudomallei* can escape from the Rab32-positive phagosome into the cytosol in a process which is largely uncharacterized but likely involves the TTSS.

Accumulating evidence suggests that in phagocytic cells, the endosomal-lysosomal degradative pathway plays a critical role in innate host-defense mechanisms against a variety of bacterial invaders by facilitating phagosome-lysosome fusion [42–46]. Here, we demonstrate that Rab32 is involved in host defense against *B*. *pseudomallei* in the early stages of infection by regulating phagosome maturation in macrophages. Specifically, we reveal that the underlying mechanism for upregulated Rab32 expression after exposure to *B*. *pseudomallei* in macrophages is through decreased miR-30b/30c expression. Subsequently, Rab32 is recruited to the *B*. *pseudomallei*–containing phagosomes with late endocytic features and it promotes the fusion of the phagosome with lysosomes to activate lysosomal acid hydrolases, thus limiting the intracellular growth of *B*. *pseudomallei* in macrophages. In addition, increasing evidence suggests that *B*. *pseudomallei* type III protein secretion system (TTSS) is crucial for vesicle escape before the bacteria can be degraded [22, 47, 48]. Thus, we speculated that there may be several TTSS effectors that interfere with Rab32 function in order to facilitates *B*. *pseudomallei* escape from the Rab32-positive compartments as an alternate fate of this pathogen (Fig 8).

Investigating the intracellular membrane trafficking involved in this host-pathogen interaction has recently led to the discovery of a novel host-defense pathway, where Rab GTPases play a central role. [49]. Previous studies indicate that Rab GTPases are involved in the infection process of many microbial pathogens. For instance, Rab5a is specifically recruited on *Leishmania donovani* phagosomes and inhibits the transport to lysosomes in human macrophages [50], Rab34 is involved in the process of mycobacterial killing by macrophages [31], and a number of Rab subfamily members, such as Rab5, Rab7, Rab14, Rab20, and Rab29 are involved in the intracellular replication of the bacterial pathogens *Mycobacterium tuberculosis* [51], *Candida albicans* [32], and *Salmonella enterica* serovar Typhimurium [52]. Recent studies also demonstrate the importance of a cell-autonomous, Rab32-dependent host-defense pathway against *Salmonella enterica* serovar Typhi and *Listeria monocytogenes* [26, 27, 53]. However, although Rab32 has been found to be crucial for suppressing the growth of the intracellular pathogens, its involvement in intracellular bacterial killing by macrophages has not yet demonstrated for *B*. *pseudomallei* and the exact mechanism underlying the Rab32-dependent elimination of bacterial pathogens is still relatively unknown.

Phagosome maturation is a complex multi-step process. The nascent phagosome develops into an acidic, protease-rich phagolysosome through a series of fission and fusion processes with endocytic organelles, a key process enabling intracellular microbial killing and degradation [54]. Therefore, it is not surprising that the trafficking of intracellular pathogens is also the site where the immune system of the host cell initiates its response. In this study, we investigated in detail the early infection events of *B*. *pseudomallei* intracellular life with respect to the phagolysosomal pathway. Our study clearly shows that Rab32 is upregulated and recruited to the *B*. *pseudomallei*-containing phagosomes in macrophages upon live *B*. *pseudomallei* infection. This is consistent with the previous reports of Rab32 being efficiently recruited to the *S*. Typhi-containing phagosomes [27, 53]. Interestingly, heat-killed *B*. *pseudomallei* fails to induce the expression and recruitment of Rab32 into the bacteria-containing phagosomes, suggesting that the Rab32 response to *B*. *pseudomallei* is may be activated by pathogen-induced damage. However, the signaling pathways underlying these differential responses still need to be identified and characterized in future studies. These observations suggest that Rab32 is a critical protective host cellular response to live *B*. *pseudomallei* infection.

MiRNAs have been described as important modulators of the host immune response against pathogenic infection, but their role in the *B*. *pseudomallei*-macrophage interplay remain largely unclear. In our previous studies, we have shown that miR-4458, miR-4667-5p, and miR-4668-5p regulate autophagy-mediated elimination of *B*. *pseudomallei* by targeting ATG10 [19]. Recently, several studies have found that miRNAs can also regulate the expression of Rab GTPases, such as Rab5a, Rab5c, and Rab11a [50, 55, 56]. Therefore, involvement of miRNAs in regulating Rab GTPase undoubtedly deserves further investigation. In this study, macrophages infected with *B*. *pseudomallei* showed a significant change in the expression pattern of a large number of miRNAs, suggesting their potential role in modulating the gene expression profile of the infected cells. Using target prediction and pathway enrichment analyses, we identified the key cellular pathways associated with the differentially expressed miRNAs and predicted mRNA targets during *B*. *pseudoamllei* infection, including the immune system, proinflammatory processes, apoptosis, cell cycle, and DNA replication and repair. Importantly, miRNA profiling revealed that four members of the miR-30 family are significantly downregulated in *B*. *pseudomallei*-infected macrophages. We further verified microarray data by qRT-PCR and found that only the expression of miR-30b and miR-30c were downregulated whereas miR-30d and miR-30e were not significantly altered. The miR-30 family members are expressed by genes localized in different genomic positions. In addition, they share a common seed sequence near the 5′ end but possess different compensatory sequences near the 3′ end [34]. These different compensatory sequences allow miR-30 family members to target different genes and pathways, thus are often differentially expressed and regulated during biological processes. Wang et al. reported that the expression levels of miR-30c, miR-30d and miR-30e were significantly decreased whereas the levels of miR-30a and miR-30b were not altered during osteoblast differentiation [57]. Therefore, the differentially regulated expression pattern with the miR-30 family members during *B*. *pseudomallei* infection could be due to differences in sequence outside the seed region. However, the exact reason for these differences still needs to be investigated further.

Rab32 has been predicted to be a direct target of miR-30 family members in previous studies [58, 59]. However, the function of miR-30b/30c on Rab32 during *B*. *pseudomallei* infection has not been reported. Indeed, we found that Rab32 is predicted as a target for miR-30 family members amongst the transcripts of all downregulated miRNAs. And there is an inverse correlation between the expression levels of miR-30b/30c and Rab32. We found that the enforced expression of miR-30b/30c suppressed Rab32 protein expression, whereas transfection of the cells with inhibitors of miR-30b/30c resulted in an increase in Rab32 expression in RAW264.7 cells or BMDMs. Additionally, a role for miR-30b/30c in Rab32 suppression was also shown in the inhibition of Rab32 recruitment to the *B*. *pseudomallei*-containing phagosomes.

We further investigated the functional significance of Rab32 upregulation and recruitment to the *B*. *pseudomallei*-containing phagosomes in infected macrophages. Previous studies have demonstrated that following active Rab5 dissociation from a phagosome, Rab7 is recruited into the late endosomal compartment, modulating its maturation [60]. Indeed, we found that *B*. *pseudomallei* recruits and retains Rab7 (a late endosome marker) but not Rab5 and EEA1 (early endosome markers) on its phagosomes. In addition, we found that *B*. *pseudomallei* not only specifically recruits Rab32 on bacterial phagosomes but also retains them in a compartment with late endocytic features, positive for LAMP1, LAMP2, and Lysotracker. Given the Rab GTPases has been demonstrated to regulate the fusion of phagosomes with lysosomes. Therefore, retention of Rab32 on *B*. *pseudomallei*-containing phagosomes might promote the constitutive fusion of bacterial phagosomes with lysosomes. We observed that the knockdown of Rab32 caused a significant decrease in the association of LAMP1 and Lysotracker with *B*. *pseudomallei*-containing phagosomes. For further evaluation of phagosome maturation, we determined the degree of phagosomal acidification and the recruitment of cathepsin D to the phagosome, because both events were critical importance for the antimicrobial activity of macrophages [61, 62]. Our results have shown that the association of both Lysotracker and CTSD with Rab32-positive phagosomes is strongly dependent on the levels of active Rab32 present in macrophages. However, we also noticed that a portion of Rab32-positive phagosomes are free of late endosomal markers (LAMP1 and LAMP2) and late endosomal/lysosomal probe (Lysotracker) from 1 to 6 h after infection (Fig 4D, 4E and 4G), suggesting that not all Rab32-positive phagosomes are fused with late endosomes. Thus, Rab32 is involved in modulating phagosome maturation, but the process by which Rab32 drive the progression from *B*. *pseudomallei*-containing phagosomes to late endosomes to degradative lysosomes is limited.

The acidic and reducing environment of lysosomes is optimal for CTSD activity. Similarly, we also demonstrated that the overexpression of EGFP-Rab32-WT or EGFP-Rab32-Q83L can enhance CTSD activation in macrophages. Altogether, these observations are consistent with the role of Rab32 in increasing the biogenesis of phagolysosomes. We also demonstrated that Rab32 activity is required for inhibiting *B*. *pseudomallei* replication in macrophages, as Rab32 knockdown or overexpression of EGFP-Rab32-T37N (inactive GDP-bound mutant) resulted in increased *B*. *pseudomallei* growth. We speculated that this was due to a defect in lysosome fusion, which ultimately disrupted the biogenesis of *B*. *pseudomallei*-containing phagolysosomes with complete degradative capacity. Previous studies have shown that intracellular survival of bacteria requires the halt of phagosome-lysosome fusion. Nonetheless, how phagosome-lysosome fusion is regulated in *B*. *pseudomallei* infection is still poorly understood. In the present study, our results demonstrate that Rab32 may regulate the delivery of *B*. *pseudomallei*-containing phagosomes to lysosomes, facilitating phagosome maturation and subsequent bacterial clearance.

Numerous studies have explored the role of miRNA regulation in the immune response against bacteria. Several deregulated miRNAs in infected host cells such as miR-146a/b, miR-155, miR-24, miR-4270, miR-27b, miR-17, miR-4458, miR-20a, and miR-144-3p have been shown to regulate cell inflammatory response, macrophage polarization, cell death/survival, and autophagy [63]. These findings indicate that the deregulation of miRNA expression may be associated with outcome of the host-pathogen interaction. Therefore, the observed effects on Rab32 expression prompted us to further explore the role of miR-30b/30c in the host immune response against *B*. *pseudomallei* infection. In this study, we found that the association of Lysotracker and CTSD with phagosomes was increased by the inhibition of miR-30b/30c expression, whereas these were inhibited by overexpression of miR-30b/30c. Additionally, inhibition of miR-30b/30c expression resulted in marked increase in the levels of mature lysosomal CTSD. Importantly, this was associated with an effective intracellular growth limitation of *B*. *pseudomallei*. Previous studies demonstrated that miR-30 family members play a crucial role in the regulation of autophagy [41, 64], and it has been reported that autophagy is capable, at least in part, to accelerate the phagosome maturation [65, 66]. However, the role of miR-30b/30c in regulation of the phagolysosomal pathway and host-defense has not been reported. In this study, we demonstrated that miR-30b/30c is associated with the modulation of phagosome maturation and affects the intracellular survival of *B*. *pseudomallei* by targeting Rab32 in host innate immune cells.

In conclusion, to the best of our knowledge, this is the novel report of miRNA-mediated Rab32 involved in modulating phagosome maturation, which at least partially exerts its antimicrobial activity by promoting phagosome maturation against *B*. *pseudomallei* infection. Our data indicate that *B*. *pseudomallei* upregulates the expression of Rab32 in infected macrophages by downregulating the expression of miR-30b/30c. Subsequently, *B*. *pseudomallei* resides, at least in the early phase of infection, in a Rab32-positive compartment, and more importantly, Rab32 promotes the fusion of *B*. *pseudomallei*-containing phagosomes with lysosomes that likely result in increased exposure of *B*. *pseudomallei* to lysosomal acid hydrolases, CTSD, and enhances the killing of *B*. *pseudomallei* by macrophages. We also demonstrate the previously unrecognized role of miR-30b/30c in modulating phagosome maturation in the host innate immune cells.

## Materials and methods

### Ethics statement

All animal experiments were performed in accordance with the Regulations for the Administration of Affairs Concerning Experimental Animals approved by the State Council of People’s Republic of China. All efforts were made to minimize animals' suffering. All studies were approved by the Laboratory Animal Welfare and Ethics Committee of the Third Military Medical University (Permit Number: SYXK-20170002).

### Cell lines and bacterial strains

Murine macrophages RAW264.7 cell line (Cat. TIB-71) and human embryonic kidney HEK293 (Cat. CRL-1573) cell line were obtained from American Type Culture Collection, Manassas, Virginia. RAW264.7 cells were grown in high glucose DMEM medium (Gibco, 11965–092) containing 10% fetal bovine serum (FBS; Gibco, 10100–147) without addition of antibiotics. HEK293 cells were routinely cultured in RPMI 1640 medium (Gibco, 11875–093) supplemented with 10% FBS and 100 U/ml penicillin/streptomycin (Gibco, 15140–122). Primary bone marrow–derived macrophages (BMDMs) were isolated from C57BL/6 mice and cultured in DMEM for 3–5 d in the presence of M-CSF (R&D Systems, 416-ML). All the above cell lines were cultured at 37°C in 5% CO2. For all experiments, the *B*. *pseudomallei* strain used in all experiments is BPC006, a virulent clinical isolate from a melioidosis patient in China[67]. And *E*. *coli* K12 (29425), *S*. *typhimurium* (14028) and *Burkholderia thailandensis* E264 (700388) were purchased from American Type Culture Collection (ATCC, Maryland, USA). Bacteria were grown in Luria-Bertani (LB) broth for 18 h at 37°C. After washing twice with phosphate buffered saline (PBS, pH 7.4; Gibco, 10010023), the number of bacteria was estimated by measuring the absorbance of the bacterial suspension at 600 nm. In general, an absorbance of 0.33 to 0.35 was equivalent to approximately 10^8^ CFU/ml of viable bacteria. The number of viable bacteria used in infection studies was determined by retrospective plating of serial 10-fold dilutions of the inoculum to LB agar. Live *B*. *pseudomallei* was handled under standard laboratory conditions (biosafety containment level 3). For experiments using heat-inactivated *B*. *pseudomallei*, bacteria were suspended in PBS, incubated at 70°C for 20 min and stored at -70°C until use.

### Antibody and reagents

The EGFP-Rab32 plasmid construct was kindly provided by Dr. Ying Wan (Biomedical Analysis Center, Army Medical University). The EGFP-Rab32-T37N and EGFP-Rab32-Q83L mutant were generated by PCR mutagenesis from the EGFP-Rab32 plasmid. The primary antibody used in this work as follows: Mouse anti-Rab32 (sc-390178) and anti-cathepsin D (sc-377299) were purchased from Santa Cruz Biotechnology. Rabbit anti-Rab5 (46449), anti-Rab7 (9367), anti-EEA1 (3288) and anti-β-actin (4970) antibody were obtained from Cell Signaling Technology. Rat anti-LAMP-1 (25245) and anti-LAMP-2 (13524) were obtained from Abcam. Mouse polyclonal anti-*B*. *pseudomallei* and rabbit polyclonal anti-*B*. *pseudomallei* antibody were obtained from immunized mice and rabbits. All secondary antibody used for immunofluorescence studies conjugated with Alexa Fluor 405, 488 and 647 were purchased from Molecular Probes, All HRP-conjugated secondary antibody (115-035-003, 111-035-003) were purchased from Jackson ImmunoResearch Laboratories.

### Western blot analysis

Samples were collected and the cell pellet was lysed in RIPA lysis buffer (50 mM Tris HCL, 150 mM NaCl, 0.1% Nonidet P-40, 0.5% sodium desoxicholate, 1% SDS, 0.5% Benzonase endonuclease (Merck Millipore) and protease and phosphatase inhibitor cocktails (Roche) for 10 min at RT and then incubated at 95°C for 5 min. Protein concentration was determined by BCA Protein Assay according to the instructions of the supplier (Thermo Fisher Scientific). Equal amounts of protein in 1x Laemmli buffer were denatured at 95°C for 5 min and subjected to standard SDS-PAGE and western blotting. A commercial protein marker was used for identification of protein size. Membranes were developed using ECL plus on ECL Hyper film (GE Healthcare), scanned, and evaluated using ImageJ. β-actin was used as loading control.

### RNA extraction and microarray experiments

Total RNA was extracted using TRIzol (Invitrogen Life Technologies) according to the manufacturer’s instruction. RNA quality was assessed by using the Agilent 2100 bioanalyzer (Agilent Technologies), and only samples with RNA integrity number >8 were used. MicroRNA microarray Assay was done using miRbase version 21.0 by LC Sciences (LC Sciences, Houston, TX). Array experiments were conducted according to the manufacturer’s instructions. Briefly, the miRNAs were labeled with Agilent miRNA labeling reagent (Agilent Technologies). Then, dephosphorylated RNA was linked with pCp-y3 and the labeled RNA was purified and hybridized to the miRNA microarray. Images were scanned with the Agilent array scanner (Agilent Technologies) using a grid file and analyzed with Agilent feature extraction software version 10.10.

GeneSpring software V12 (Agilent Technologies) was used for summarization, normalization, and quality control of miRNA microarray data. The miRNA array data were calculated by first subtracting the background value and then normalizing the signals by locally weighted regression. The express levels of miRNAs were designated as statistically significant when the 2-tailed P value was ≤0.05. And signals <500 were interpreted as false-positive result. The statistically significant messenger RNAs were selected based on the fold change and adjusted P value ≤0.05.

### Luciferase assay

Luciferase reporter construct was made by cloning mouse Rab32 sequence containing the potential miR-30b/c binding site into pMIR-Report construct (Ambion, Austin, USA). The DNA oligonucleotides containing wild-type (WT) or mutant (Mut) 3’UTR of Rab32 were synthesized with flanking *Spe* I, *Apa* I and *Hind* III restriction enzyme digestion sites, respectively. All of the sequences are shown in Supplementary information, S2 Table. The HEK-293 cells were transfected with 0.8 μg of indicated wide-type or mutant firefly luciferase reporter vectors, 100 nM indicated miRNAs mimic, inhibitor or control (RiboBio), and 0.04 μg of Renilla luciferase control vector (pRL-TK-Promega) using Lipofectamine 3000 (Invitrogen Life Technologies). After transfection for 24 h, all of the cells were lysed via dual luciferase reporter assay system (Promega), and then the fluorescence activity was detected via GloMax 20/20 Luminometer. Firefly luciferase activity was normalized to Renilla luciferase activity.

### Quantitative real-time-PCR (qRT-PCR)

qRT-PCR assays for miR-30b and miR-30c were performed by using TaqMan miRNA assays (Ambion) in a Bio-Rad IQ5 (Bio-Rad Laboratories, Inc). The reactions were performed using the following parameters: 95°C for 2 min followed by 40 cycles of 95°C for 15 s and 60°C for 30 s. U6 small nuclear RNA was used as an endogenous control for data normalization. Relative expression was calculated using the comparative threshold cycle method. Quantitative RT-PCR analyses for the mRNA of Rab32 was performed by using PrimeScript RT-PCR kits (Takara). The mRNA levels of β-actin were used as an internal control. The primers were shown in Supplementary information, S2 Table.

### Macrophage transfection

For miRNA transfections, miR-30b and miR-30c mimic, miR-30b and miR-30c inhibitor are obtained from RiboBio (Guangzhou, Guangdong, China). The sequences are as follows: miR-30b mimic, 5′-UGUAAACAUCCUACACUCAGCU-3′ and miR-30c mimic, 5′-UGUAAACAUCCUACACUCUCAGC-3′; miR-30b inhibitor, 5′-AGCUGAGUGUAGGAUGUUUACA-3′ and miR-30c inhibitor, 5′- GCUGAGAGUGUAGGAUGUUUACA-3′. mimic Negative Control, 5′- UUUGUACUACACAAAAGUACUG-3′ and inhibitor Negative Control, 5′- CAGUACUUUUGUGUAGUACAAA-3′. RAW264.7 cells or BMDMs were seeded in 24-well plates and co-transfected with miR-30b or mi-30c mimic (30 nM), inhibitor (30 nM) and NC control oligo (30 nM) using Lipofectamine 3000 (Invitrogen, L3000008) according to the manufacturer’s instructions. After 24 h, cells were harvested and the expression levels of Rab32 mRNA or protein were detected by qRT-PCR and Western blotting as described above. For siRNA transfections, RAW264.7 macrophages were seeded at 1×10^6^ per well in imaging dishes or standard 6-well culture plates for RNA or protein extraction in antibiotic-free DMEM and were incubated overnight. Cells were transfected with Lipofectamine 3000, Opti-MEM (Invitrogen, United Kingdom), and 50 nM Silencer Select Rab32 siRNA (Santa Cruz Biotechnology, 152636) for 24 h. The effects of Rab32 siRNA were compared with those of a nontargeting control siRNA (Santa Cruz Biotechnology, sc-44230). For plasmid DNA transfections, RAW264.7 macrophages were seeded at 5 × 10^5^ per well 1 d before the transfection according to the manufacturer’s protocol. Cells were transfected 16–20 h before further experiments. All experiments were performed in triplicate.

### Indirect immunofluorescence

For immunofluorescence studies, Samples were washed with PBS prior to fixation with 4% paraformaldehyde (PFA, Electron Microscopy Sciences) for 10 min. Cells were washed three times with PBS and permeablized with 0.05% Saponin (Sigma), 1% bovine serum albumin (BSA, Sigma-Aldrich) in PBS for 10 min at room temperature. Subsequently, samples were incubated in 1% BSA/PBS for 5 min prior to incubation with primary and secondary antibody in 1% BSA/PBS for 1 h. Three washing steps with PBS for 5 min followed each antibody incubation. Finally, the nuclear stain DAPI (Life technologies, 300 nM) was applied for 10 min at room temperature. Glass coverslips were mounted on glass slides (Thermo Scientific) using Fluorescent mounting medium (Dako Cytomation). For analysis of association of acidic compartments with *B*. *pseudomallei* phagosomes. RAW264.7 or BMDMs were incubated with 50 nM Lysotracker-DND99 (Molecular Probes, L7528) for 1 h prior to infection. The cells viewed using a laser-scanning confocal microscope (Zeiss, Germany).

### Transmission electronic microscopy

RAW264.7 cells were treated as indicated and were fixed in 2.5% glutaraldehyde at 4°C overnight and postfixed with 2% osmium tetroxide for 1.5 h at room temperature. After fixation, cells were embedded and stained with uranyl acetate/lead citrate. The sections were examined under a transmission electron microscope (JEM-1400PLUS, Japan) at 60 kV.

### Intracellular survival of bacteria

Bacterial invasion of RAW264.7 cells or BMDMs was investigated by using the method described by Elsinghorst, except for the following modifications [68]. Cells were infected with *B*. *pseudomallei* at an MOI of 10:1. One hour after infection, cells were washed twice with phosphate-buffered saline (PBS), and 2 ml of fresh culture medium containing 250 μg of kanamycin per ml was added, and the preparation was incubated to kill the extracellular bacteria. After the indicated time points, cells were washed three times with PBS and lysed with 1 ml of 0.1% Triton X-100 (Sigma) after infection. Diluted cell lysates were plated on Luria broth plates. Colonies were counted after 36 h. Experiments were performed at least three times in triplicates.

### Quantification of *B*. *pseudomallei* internalization

Macrophages silenced for Rab32 or expressing control siRNA, or transfected with pEGFP, pEGFP-Rab32, pEGFP-Rab32-T37N and pEGFP-Rab32-Q83L were incubated with *B*. *pseudomallei* for 0.5 h, washed, and chased for 0.5 h as described and processed for imaging. Confocal images were made from consecutive fields, until 100 transfected cells were imaged. The transfected cell containing *B*. *pseudomallei* were counted and divided by the total number of the imaged cells.

### Statistical analysis

All images were analyzed by ImageJ software (MD, USA). Images of the samples were acquired with blinding of the experimental conditions. The association of different markers with *B*. *pseudomallei* was measured by automated analysis of the mean relative fluorescent marker intensity in a 2-pixel wide ring around bacteria or by counting the percentage of *B*. *pseudomallei* associated with a marker. At least 250 or 100 bacteria per biological replicate were analysed *B*. *pseudomallei* the automated analysis or manual count respectively. The results are expressed as the mean ± SD of at least three separate experiments performed in triplicate. The differences between the groups were determined with the SPSS 13.0 software. Student’s t-test was used to analyze the data. The differences were considered significant at P<0.05. Statistically significant differences are indicated by asterisks (*P<0.05, **P<0.01).

## Supporting information

S1 Fig Heat-killed *B*. *pseudomallei* does not trigger the recruitment of Rab32 to phagosomes and induce the expression of Rab32. (A) RAW264.7 cells were infected with heat-killed *B*. *pseudomallei* (MOI = 10:1) and imaged at the indicated time points: 1 to 6 h and stained with anti-Rab32 antibody (green), anti-*B*. *pseudomallei* antibody (red). Images show maximum-intensity projections of confocal Z-stacks. Scale bar is 5 μm. (B and C) RAW264.7 cells were infected with live or heat-killed (HK) *B*. *pseudomallei*, at MOI of 10 for 4 h. The expression levels of Rab32 were analyzed by qRT-PCR and Western blot. (D and E) Representative images of RAW264.7 cells were infected with live or heat-killed *B*. *pseudomallei* for 2 h, and stained with anti-Rab32 antibody (green), anti-*B*. *pseudomallei* antibody (red) or DAPI (blue). Quantification showing the percentage of the cells containing *B*. *pseudomallei*. The average ± SD. is shown for three independent experiments. Scale bar is 10 μm. (*P<0.05, **P<0.01). *ns*, no significant difference.
(TIF)

S2 Fig Heat-killed *B*. *pseudomallei* have no significant effects on the expression levels of miR-30 family members. **(A and B)** Confirmation of microarray results by qRT-PCR. qRT-PCR analysis of the expression levels of miR-30b, miR-30c, miR-30d, and miR-30e in RAW264.7 cells infected with heat-killed *B*. *pseudomallei* (MOI = 10) for 0, 1, 2, 4, 6, and 8 h, or at MOI = 0, 1, 10, 20, 50, and 100 for 4 h. Experiments performed in triplicates showed consistent results.
(TIF)

S3 Fig The expression of intracellular miR-30b and miR-30c after transfected with the miRNA control, mimic, or inhibitor. **(A and B)** After transfected with miRNAs control, mimic or inhibitor for 24 h, the expression of miR-30b and miR-30c was performed by using TaqMan miRNA assays. Data are representatives of at least three independent experiments, * P< 0.05, ** P< 0.01.
(TIF)

S4 Fig Localization analysis of the late endosomal markers in *B*. *pseudomallei* phagosomes. **(A-C)** RAW264.7 cells were infected with *B*. *pseudomallei*, at an MOI of 10 for indicated time point. Cells were stained with anti-EEA1, anti-Rab5, and anti-Rab7 antibodies (green), or anti-*B*. *pseudomallei* antibody (red) and colocalization was determined by confocal microscopy. Scale bar is 5 μm. **(D and E)** RAW264.7 cells expressing EGFP-Rab32 were infected with *B*. *pseudomallei* for indicated time point, afterwards cells were subjected to immunofluorescence for LAMP1 or LAMP2 (red) and stained with an anti-*B*. *pseudomallei* antibody (blue). Scale bar is 5 μm. **(F)** RAW264.7 cells expressing EGFP-Rab32 were incubated with 50 nM Lysotracker (red) for 1 h before infection with *B*. *pseudomallei* for indicated time point. Cells were stained with anti-*B*. *pseudomallei* antibody (blue) and colocalization was determined by confocal microscopy. Scale bar is 5 μm. All results are representative of three independent observations.
(TIF)

S5 Fig Rab32 knockdown or overexpression does not affect not affect phagocytosis the internalization rate of *B*. *pseudomallei*. **(A)** RAW264.7 cells were transfected with Rab32 siRNA or control siRNA for 24 h, then infected with *B*. *pseudomallei* at an MOI of 10:1 for 1 h. Quantification showing the percentage of the cells containing *B*. *pseudomallei*. **(B)** Quantification of the total percentage of cells containing *B*. *pseudomallei*, comparing RAW264.7 cells transfected with pEGFP, pEGFP-Rab32 WT, pEGFP-Rab32 T37N or pEGFP-Rab32 Q83L. The numbers of internalization bacteria were quantified in confocal microscopic images. Approximately 200–300 cells were sequentially sampled for each experiment. The data shown represents the mean value ± SD based on three independent experiments. *ns*, no significant difference.
(TIF)

S6 Fig EGFP-Rab32-WT and its GTP-bound mutant EGFP-Rab32-Q83L increase the recruitment of Rab32 to *B*. *pseudomallei*-containing phagosomes. **(A)** RAW264.7 cells were transfected with pEGFP, pEGFP-Rab32-WT, pEGFP-Rab32-T37N or pEGFP-Rab32-Q83L, and 24 h later, cells were infected with *B*. *pseudomallei* (MOI = 10: 1). The infected RAW264.7 cells were stained with anti-*B*. *pseudomallei* antibodies (red) and DAPI (blue). Scale bar is 5μm. **(B)** Quantification showing the percentage of association of EGFP-Rab32 to *B*. *pseudomallei* containing phagosomes. Data show mean ± SD of the percentage of bacteria recovered compared with control cells from two independent experiments. (*P<0.05, **P<0.01). *ns*, no significant difference.
(TIF)

S7 Fig miR-30b and miR-30c modulates Rab32 expression. **(A and B)** BMDMs were transiently transfected with miR30b mimic, miR30c mimic, miR30b inhibitor, miR30c inhibitor or control for 24 h. The mRNA and protein level of Rab32 were determined by qRT-PCR and western blot. Data are representative of three independent experiments (** P< 0.01).
(TIF)

S1 Table Differentially expressed microRNAs between *Burkholderia pseudomallei* infected and uninfected cells. (DOCX)

S2 Table Sequences of DNA oligonucleotides and primers used in the paper. (DOCX)
