# Supplementary figures and images for: Rab32 GTPase, as a direct target of miR-30b/c, controls the intracellular survival of Burkholderia pseudomallei by regulating phagosome maturation
Source: PLoS Pathog. 2019 Jun 14;15(6):e1007879. doi: 10.1371/journal.ppat.1007879 (PMC6594657; doi:10.1371/journal.ppat.1007879)

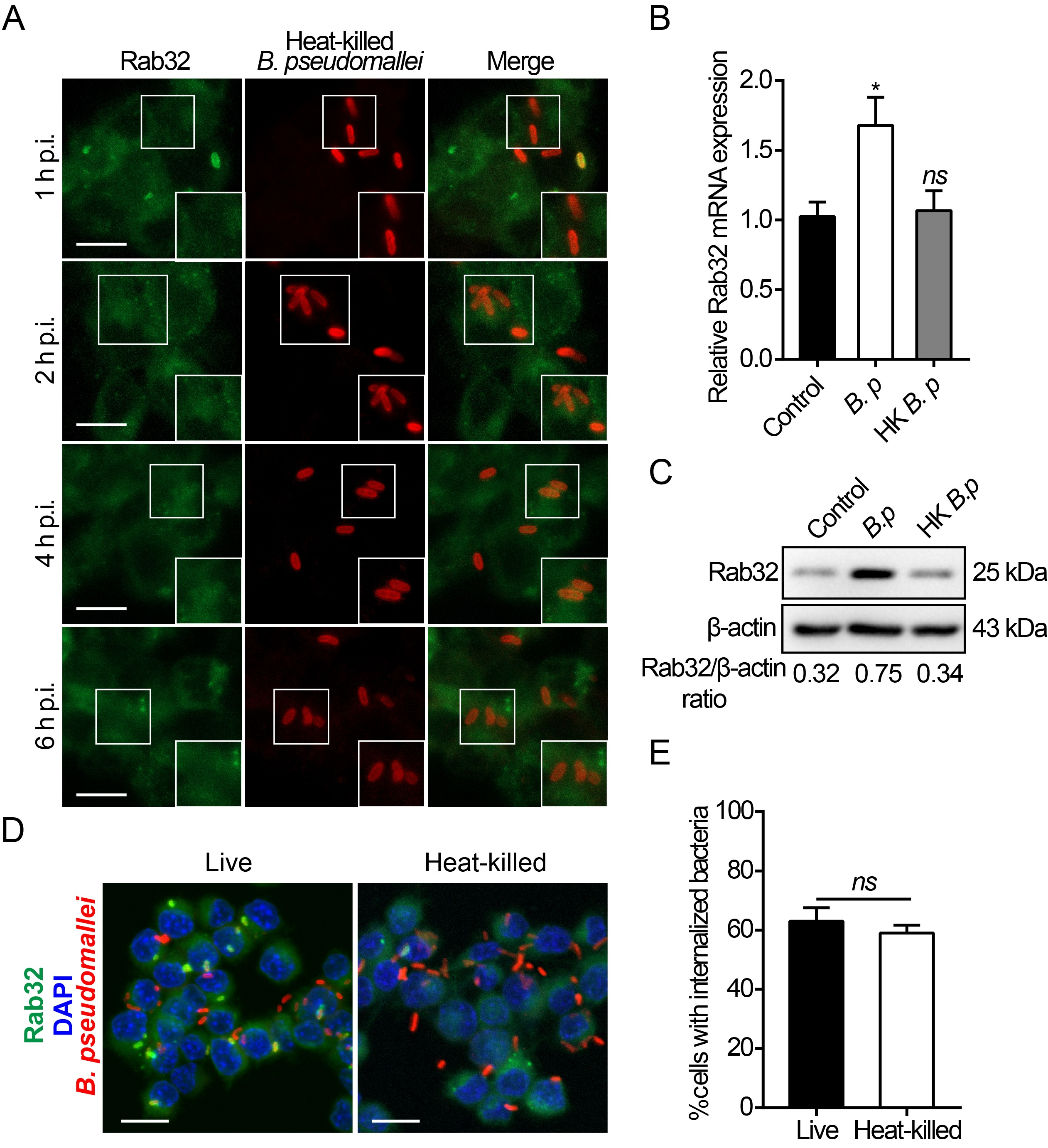

Supplement: S1 Fig — (A) RAW264.7 cells were infected with heat-killed B. pseudomallei (MOI = 10:1) and imaged at the indicated time points: 1 to 6 h and stained with anti-Rab32 antibody (green), anti-B. pseudomallei antibody (red). Images show maximum-intensity projections of confocal Z-stacks. Scale bar is 5 μm. (B and C) RAW264.7 cells were infected with live or heat-killed (HK) B. pseudomallei, at MOI of 10 for 4 h. The expression levels of Rab32 were analyzed by qRT-PCR and Western blot. (D and E) Representative images of RAW264.7 cells were infected with live or heat-killed B. pseudomallei for 2 h, and stained with anti-Rab32 antibody (green), anti-B. pseudomallei antibody (red) or DAPI (blue). Quantification showing the percentage of the cells containing B. pseudomallei. The average ± SD. is shown for three independent experiments. Scale bar is 10 μm. (*P<0.05, **P<0.01). ns, no significant difference. (TIF) [file ppat.1007879.s001.tif]

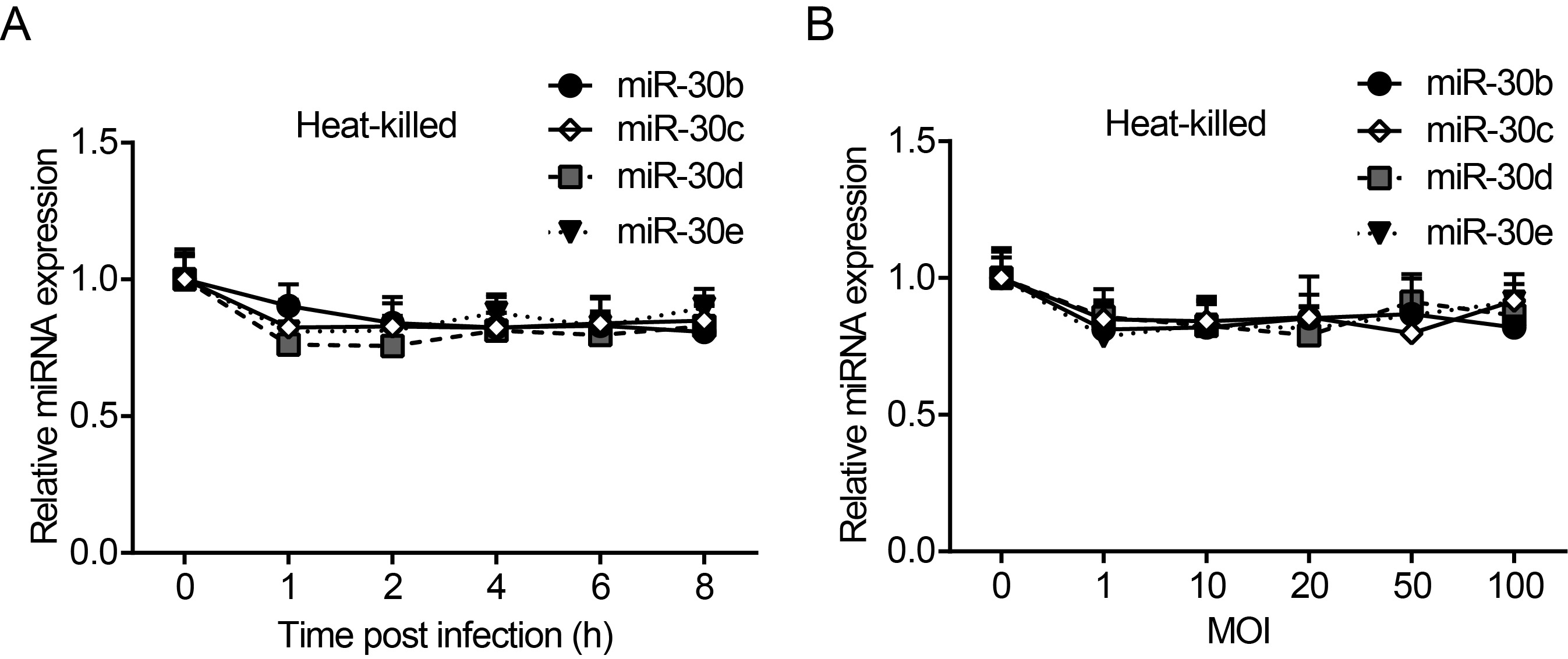

Supplement: S2 Fig — (A and B) Confirmation of microarray results by qRT-PCR. qRT-PCR analysis of the expression levels of miR-30b, miR-30c, miR-30d, and miR-30e in RAW264.7 cells infected with heat-killed B. pseudomallei (MOI = 10) for 0, 1, 2, 4, 6, and 8 h, or at MOI = 0, 1, 10, 20, 50, and 100 for 4 h. Experiments performed in triplicates showed consistent results. (TIF) [file ppat.1007879.s002.tif]

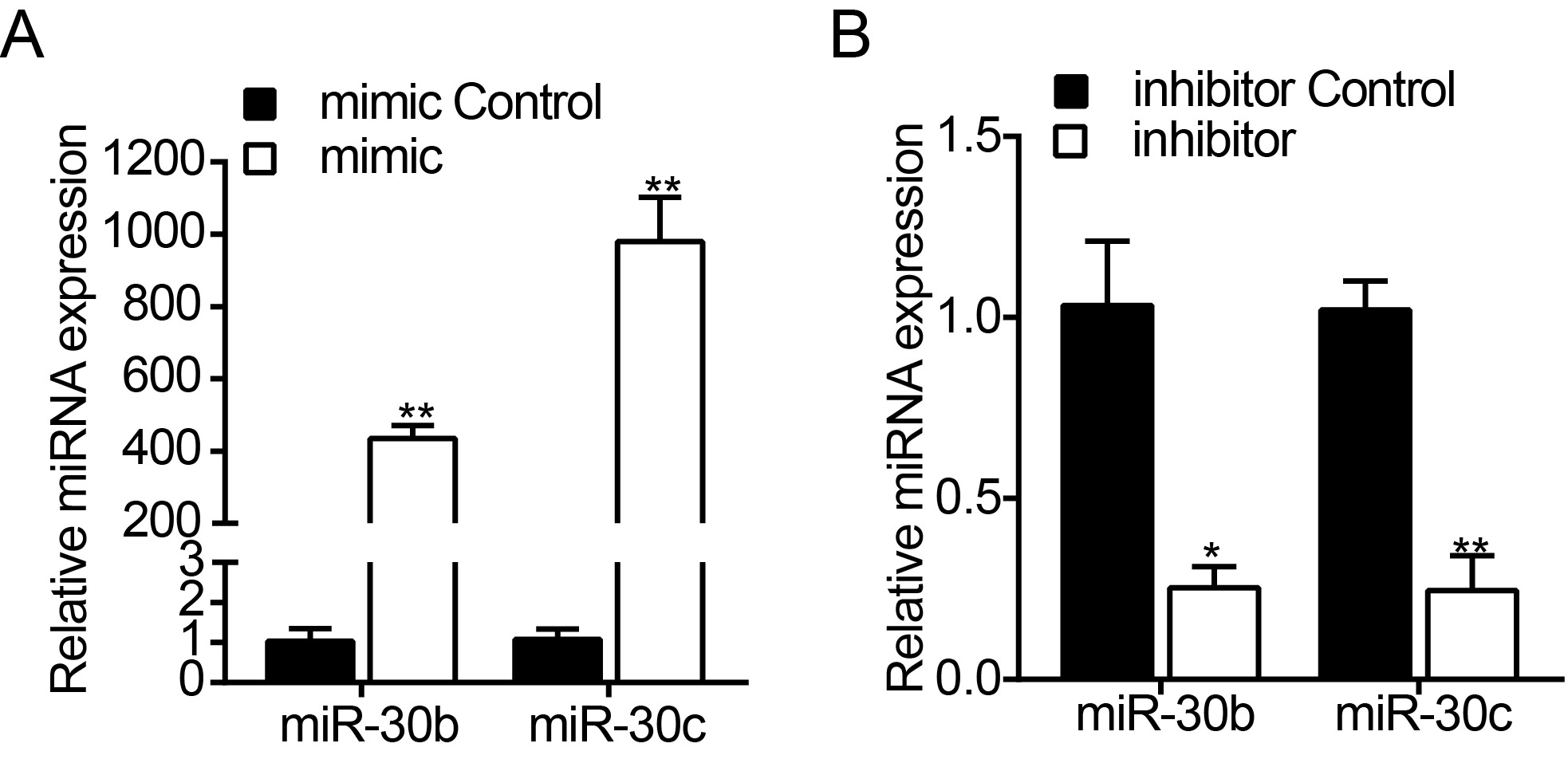

Supplement: S3 Fig — (A and B) After transfected with miRNAs control, mimic or inhibitor for 24 h, the expression of miR-30b and miR-30c was performed by using TaqMan miRNA assays. Data are representatives of at least three independent experiments, * P< 0.05, ** P< 0.01. (TIF) [file ppat.1007879.s003.tif]

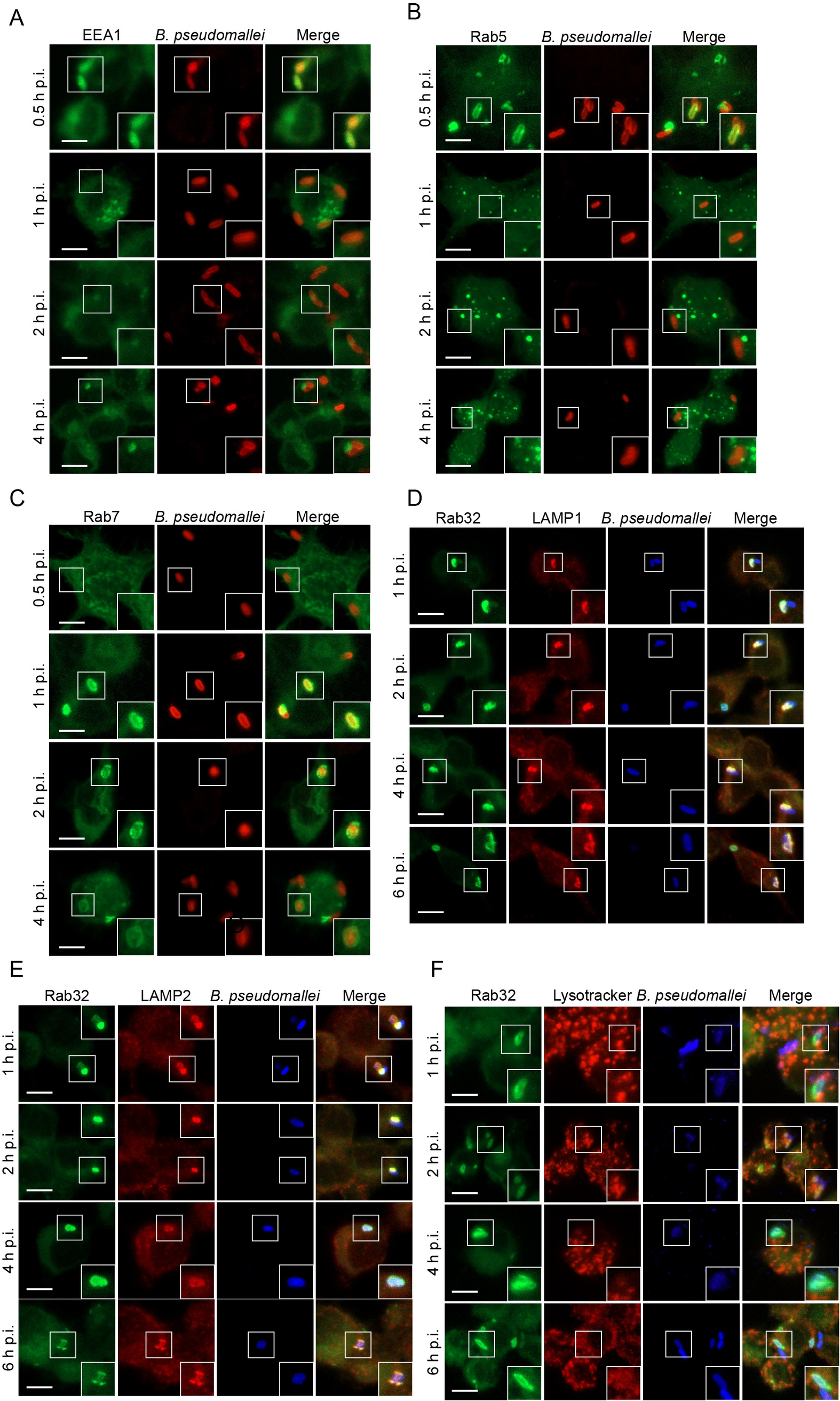

Supplement: S4 Fig — (A-C) RAW264.7 cells were infected with B. pseudomallei, at an MOI of 10 for indicated time point. Cells were stained with anti-EEA1, anti-Rab5, and anti-Rab7 antibodies (green), or anti-B. pseudomallei antibody (red) and colocalization was determined by confocal microscopy. Scale bar is 5 μm. (D and E) RAW264.7 cells expressing EGFP-Rab32 were infected with B. pseudomallei for indicated time point, afterwards cells were subjected to immunofluorescence for LAMP1 or LAMP2 (red) and stained with an anti-B. pseudomallei antibody (blue). Scale bar is 5 μm. (F) RAW264.7 cells expressing EGFP-Rab32 were incubated with 50 nM Lysotracker (red) for 1 h before infection with B. pseudomallei for indicated time point. Cells were stained with anti-B. pseudomallei antibody (blue) and colocalization was determined by confocal microscopy. Scale bar is 5 μm. All results are representative of three independent observations. (TIF) [file ppat.1007879.s004.tif]

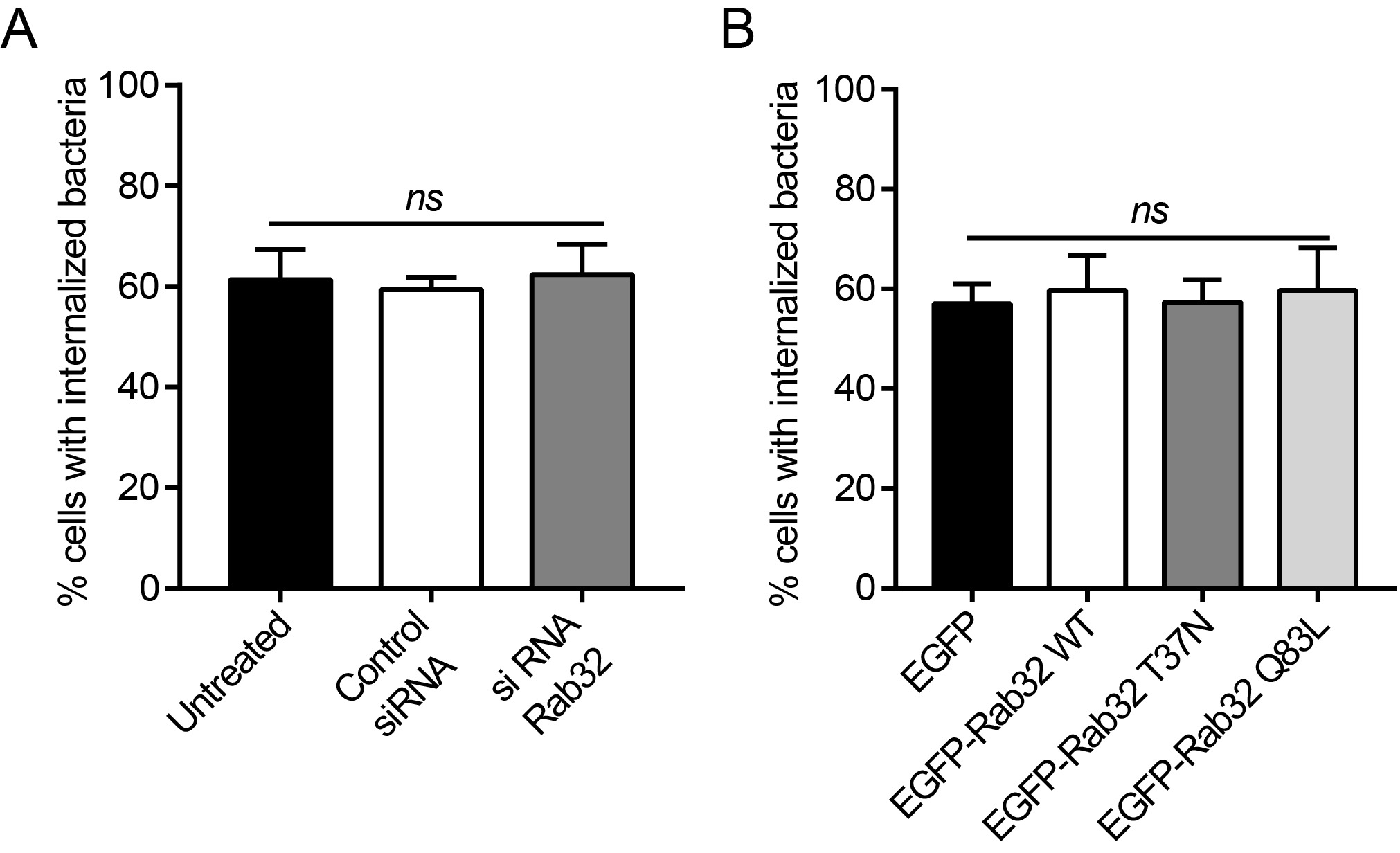

Supplement: S5 Fig — (A) RAW264.7 cells were transfected with Rab32 siRNA or control siRNA for 24 h, then infected with B. pseudomallei at an MOI of 10:1 for 1 h. Quantification showing the percentage of the cells containing B. pseudomallei. (B) Quantification of the total percentage of cells containing B. pseudomallei, comparing RAW264.7 cells transfected with pEGFP, pEGFP-Rab32 WT, pEGFP-Rab32 T37N or pEGFP-Rab32 Q83L. The numbers of internalization bacteria were quantified in confocal microscopic images. Approximately 200–300 cells were sequentially sampled for each experiment. The data shown represents the mean value ± SD based on three independent experiments. ns, no significant difference. (TIF) [file ppat.1007879.s005.tif]

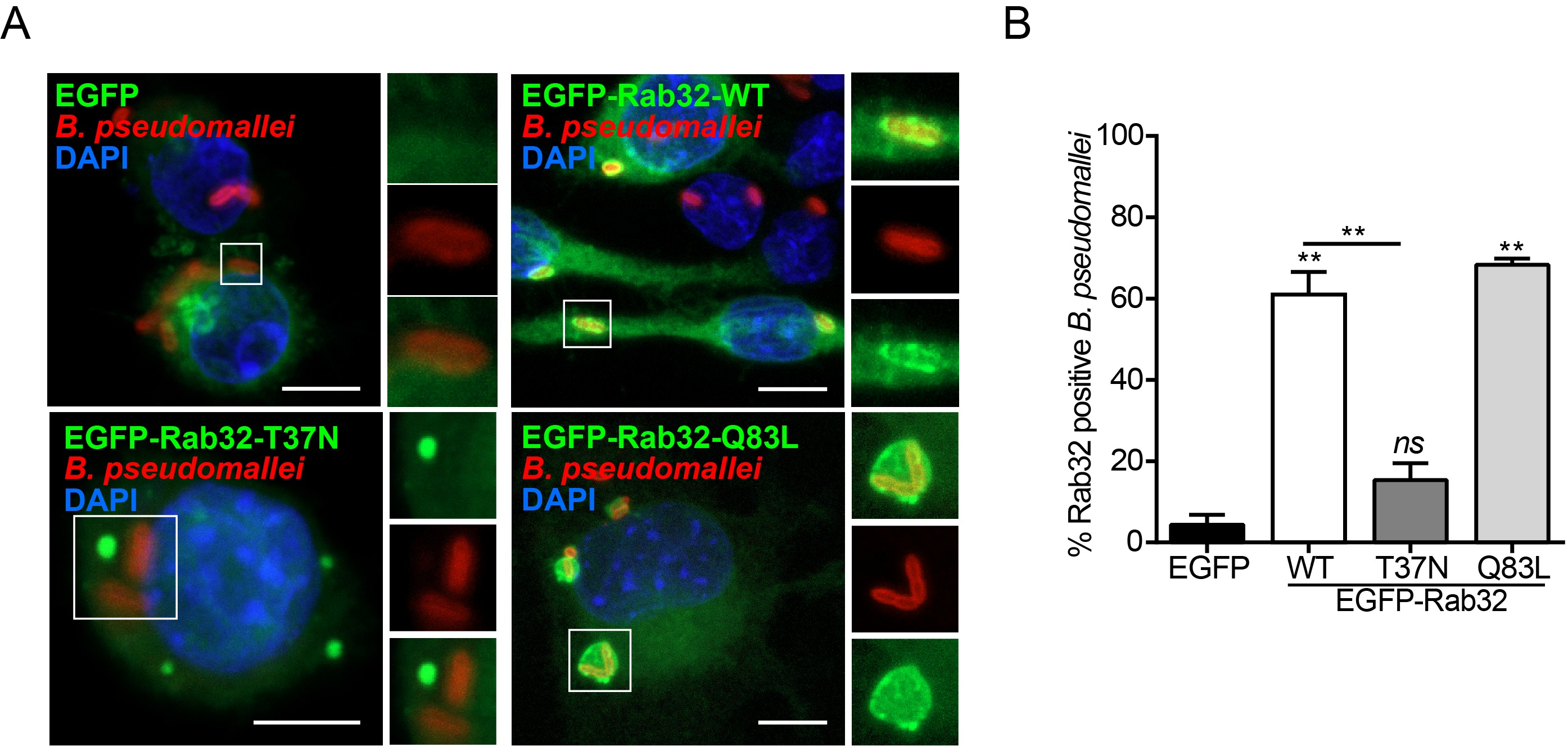

Supplement: S6 Fig — (A) RAW264.7 cells were transfected with pEGFP, pEGFP-Rab32-WT, pEGFP-Rab32-T37N or pEGFP-Rab32-Q83L, and 24 h later, cells were infected with B. pseudomallei (MOI = 10: 1). The infected RAW264.7 cells were stained with anti-B. pseudomallei antibodies (red) and DAPI (blue). Scale bar is 5μm. (B) Quantification showing the percentage of association of EGFP-Rab32 to B. pseudomallei containing phagosomes. Data show mean ± SD of the percentage of bacteria recovered compared with control cells from two independent experiments. (*P<0.05, **P<0.01). ns, no significant difference. (TIF) [file ppat.1007879.s006.tif]

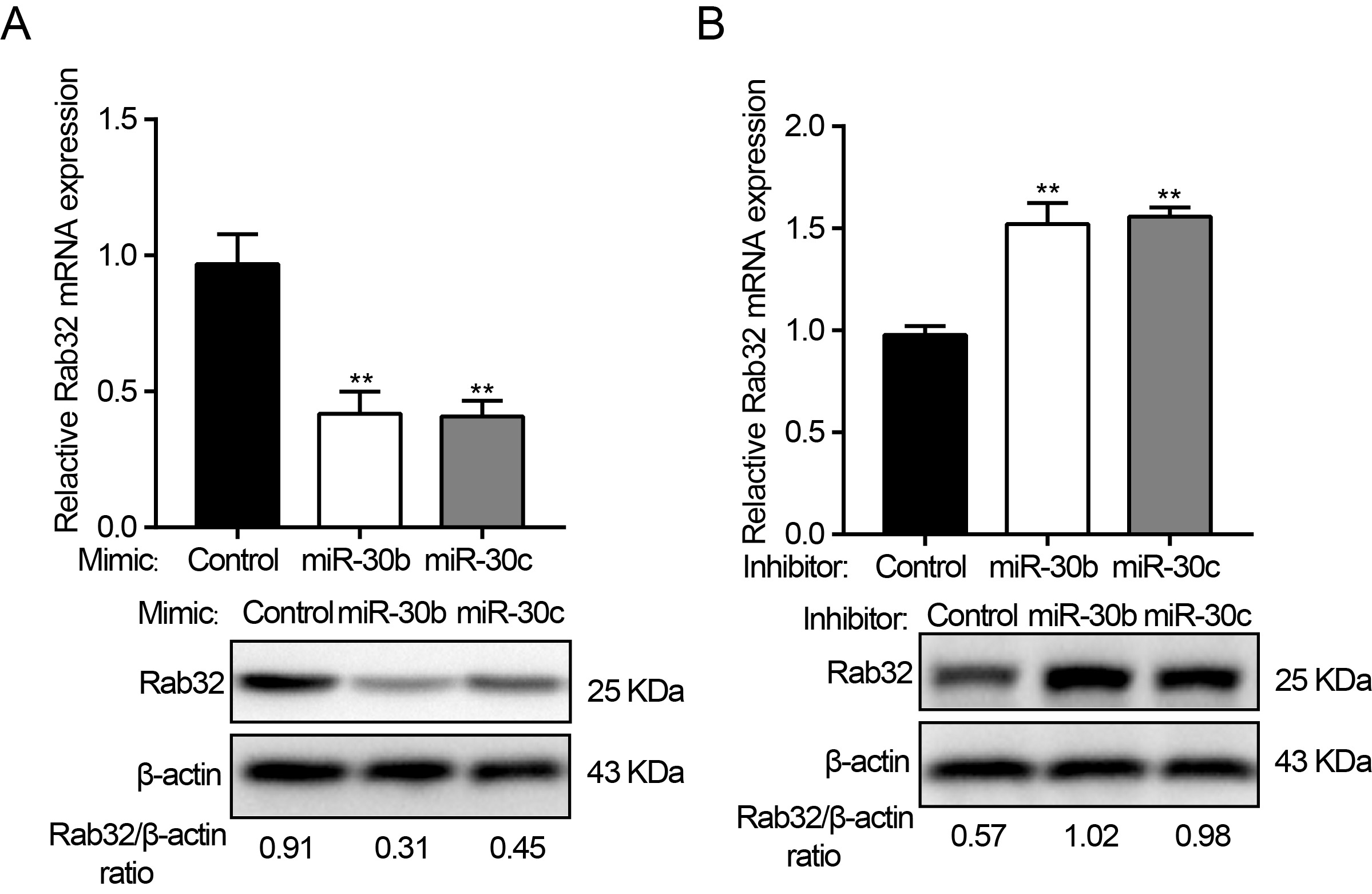

Supplement: S7 Fig — (A and B) BMDMs were transiently transfected with miR30b mimic, miR30c mimic, miR30b inhibitor, miR30c inhibitor or control for 24 h. The mRNA and protein level of Rab32 were determined by qRT-PCR and western blot. Data are representative of three independent experiments (** P< 0.01). (TIF) [file ppat.1007879.s007.tif]
