# Supplementary material for: Rab32 GTPase, as a direct target of miR-30b/c, controls the intracellular survival of Burkholderia pseudomallei by regulating phagosome maturation
Source: PLoS Pathog. 2019 Jun 14;15(6):e1007879. doi: 10.1371/journal.ppat.1007879 (PMC6594657; doi:10.1371/journal.ppat.1007879)
Supplement: S1 Table — (DOCX) [file ppat.1007879.s008.docx]

**S1 Table. Differentially expressed microRNAs between *Burkholderia pseudomallei* infected and uninfected Cells**

|  |  |  | **Uninfected** | | | ***B. pseudomallei*** | | |
| --- | --- | --- | --- | --- | --- | --- | --- | --- |
| **microRNA** | **Log_2_ (FC)** | **Regulation** | **NC-1** | **NC-2** | **NC-3** | **Infected-1** | **Infected-2** | **Infected-3** |
| mmu-miR-574-5p | -1.9355302 | down | -5.33104 | -3.96846 | -3.48304 | -6.6916 | -6.69899 | -6.6219 |
| mmu-miR-690 | -1.5835631 | down | -3.88956 | -2.65595 | -3.00131 | -5.2598 | -5.28162 | -5.17474 |
| mmu-miR-712 | -1.5233064 | down | -6.23101 | -6.18858 | -5.69338 | -7.4568 | -7.78443 | -7.35831 |
| mmu-miR-3968 | -1.3849916 | down | 1.847949 | 2.319874 | 2.162612 | 0.1521 | 0.060102 | 0.187152 |
| mmu-miR-1839-3p | -1.357717 | down | -5.33046 | -4.7393 | -4.94806 | -6.8305 | -6.81945 | -6.87577 |
| mmu-miR-30c | -1.3434536 | down | -4.35628 | -3.4054 | -3.59913 | -5.4658 | -5.5326 | -5.46124 |
| mmu-miR-202-3p | -1.220464 | down | -3.38911 | -2.89971 | -2.81562 | -4.7016 | -4.79302 | -4.35972 |
| mmu-miR-5117 | -1.2155886 | down | -5.06105 | -4.67404 | -4.51607 | -6.4613 | -6.61916 | -6.27954 |
| mmu-miR-466i-5p | -1.1793573 | down | -3.5304 | -2.32928 | -1.90664 | -3.9495 | -4.01129 | -3.83719 |
| mmu-miR-30b | -1.1359885 | down | -3.2777 | -2.54884 | -2.82261 | -4.316 | -4.30903 | -4.32522 |
| mmu-miR-146b | -0.9822948 | down | -3.82917 | -3.55943 | -3.26706 | -4.7334 | -4.95394 | -4.59349 |
| mcmv-miR-M23-1-5p | -0.98007965 | down | -4.99489 | -4.84051 | -5.00623 | -6.2514 | -6.49557 | -6.17499 |
| mmu-miR-17 | -0.94423485 | down | -4.21005 | -3.96975 | -4.11974 | -5.4074 | -5.56023 | -5.28131 |
| mmu-miR-500 | -0.8755388 | down | -5.37138 | -5.06011 | -5.3494 | -6.4215 | -6.39221 | -6.49407 |
| mmu-miR-182 | -0.86771536 | down | -4.39393 | -3.85906 | -4.31022 | -5.3034 | -5.26683 | -5.3583 |
| mmu-miR-423-5p | -0.8392134 | down | -5.28819 | -5.10666 | -5.31923 | -6.4493 | -6.43774 | -6.45818 |
| mmu-miR-155 | -0.83578205 | down | -1.83926 | -1.59879 | -1.24064 | -2.4331 | -2.77882 | -2.41517 |
| mmu-miR-101a | -0.8189812 | down | -5.30309 | -5.02997 | -5.72645 | -6.6564 | -6.48756 | -6.7818 |
| mmu-miR-30e | -0.81565404 | down | -4.1273 | -3.56258 | -3.84334 | -4.8949 | -4.89244 | -4.89678 |
| mmu-miR-3963 | -0.81476164 | down | 4.878017 | 5.27137 | 5.262724 | 4.1283 | 3.897613 | 4.168403 |
| mmu-miR-331-3p | -0.8022108 | down | -5.23951 | -5.12348 | -5.22981 | -6.4069 | -6.43986 | -6.29259 |
| mmu-miR-3069-3p | -0.80196047 | down | -4.31739 | -4.05293 | -4.30594 | -5.3075 | -5.37517 | -5.30376 |
| mmu-miR-494 | -0.7821398 | down | -3.04046 | -2.64358 | -2.47907 | -3.7867 | -3.8263 | -3.55869 |
| mmu-miR-222 | -0.7811744 | down | -2.42248 | -1.86623 | -2.09523 | -3.0037 | -3.11693 | -2.97198 |
| mmu-miR-142-3p | -0.764465 | down | 0.217917 | 0.59461 | 0.345629 | -0.7247 | -0.68338 | -0.79348 |
| mmu-miR-128 | -0.7632475 | down | -5.22711 | -5.12287 | -5.3805 | -6.3667 | -6.35421 | -6.48744 |
| mmu-miR-374c | -0.7440109 | down | -4.81168 | -4.77667 | -4.86723 | -6.082 | -6.10091 | -5.99541 |
| mmu-miR-5105 | -0.7090802 | down | -5.04622 | -4.57818 | -4.74904 | -5.7633 | -5.87655 | -5.65513 |
| mmu-miR-30d | -0.70287514 | down | -4.47444 | -3.93651 | -4.02378 | -5.0536 | -5.08163 | -4.9289 |
| mmu-miR-26a | -0.7026088 | down | -4.00441 | -3.39615 | -3.89734 | -4.6935 | -4.53891 | -4.83472 |
| mmu-miR-221 | -0.69274366 | down | -0.28281 | 0.30893 | 0.07475 | -0.8125 | -0.75908 | -0.8311 |
| mmu-miR-223 | -0.67982674 | down | -4.48384 | -3.63554 | -4.07212 | -4.6881 | -4.66773 | -4.9399 |
| mmu-miR-222* | -0.6732969 | down | -7.64585 | -6.33975 | -6.14387 | -7.3457 | -7.34786 | -7.24402 |
| mmu-miR-125a-5p | -0.6570287 | down | -3.65686 | -3.32997 | -3.39073 | -4.268 | -4.3262 | -4.23078 |
| mmu-miR-3072* | -0.6464944 | down | -5.57614 | -5.33354 | -5.33007 | -6.1659 | -6.5808 | -6.01003 |
| mmu-miR-101b | -0.6434951 | down | -5.30054 | -4.968 | -5.32711 | -6.1228 | -6.06728 | -6.245 |
| mmu-miR-1224 | -0.6394143 | down | -2.1914 | -1.42175 | -1.51686 | -2.4494 | -2.55246 | -2.4131 |
| mmu-miR-365 | -0.63373613 | down | -3.13757 | -3.01229 | -3.03343 | -3.8928 | -4.08302 | -3.84537 |
| mmu-miR-193 | -0.6306491 | down | -4.71135 | -4.50151 | -4.58307 | -5.3952 | -5.61767 | -5.32704 |
| mmu-miR-338-3p | -0.6299219 | down | -6.27055 | -5.21836 | -6.2851 | -6.6868 | -6.4986 | -6.83965 |
| mmu-miR-125b-5p | -0.62836975 | down | -0.64338 | -0.37608 | -0.70053 | -1.4821 | -1.46612 | -1.49895 |
| mmu-miR-5109 | -0.62689114 | down | -0.21748 | 0.420893 | 0.478437 | -0.4083 | -0.71651 | -0.36889 |
| mmu-miR-3473b | -0.6199138 | down | -3.10331 | -2.37718 | -2.31789 | -3.4185 | -3.59065 | -3.23765 |
| mmu-miR-29c | -0.6149514 | down | -2.28672 | -1.75133 | -2.25141 | -2.993 | -2.7723 | -3.04779 |
| mmu-miR-99b | -0.61349726 | down | -3.72384 | -3.44041 | -3.35744 | -4.3018 | -4.30741 | -4.19616 |
| mmu-miR-142-5p | -0.60971165 | down | -3.06519 | -2.67974 | -2.99627 | -3.7736 | -3.7562 | -3.80962 |
| mmu-miR-2137 | -0.6077666 | down | -6.42053 | -5.54077 | -5.54566 | -6.5469 | -6.58353 | -6.50239 |
| mmu-miR-345-5p | -0.59570265 | down | -5.818 | -5.39032 | -5.47484 | -6.7044 | -6.72967 | -6.17164 |
| mmu-miR-146a | -0.56862235 | down | -2.24885 | -1.68294 | -2.35657 | -2.8587 | -2.61789 | -2.96164 |
| mmu-miR-26b | -0.5482452 | down | -3.00928 | -2.58553 | -2.97716 | -3.5821 | -3.56826 | -3.68303 |
| mmu-miR-140* | -0.5349245 | down | -3.24893 | -2.90242 | -3.16781 | -3.7831 | -3.78503 | -3.7642 |
| mmu-miR-148b | -0.5136037 | down | -5.82553 | -5.33992 | -5.77187 | -6.3069 | -6.3438 | -6.24123 |
| mmu-miR-27b | -0.4914974 | down | -1.81118 | -1.33946 | -1.88376 | -2.4197 | -2.28602 | -2.43265 |
| mmu-miR-23b | -0.4899928 | down | -1.04277 | -0.6082 | -1.01339 | -1.4837 | -1.4621 | -1.54689 |
| mmu-miR-425 | -0.48720884 | down | -3.96613 | -3.74366 | -4.02277 | -4.559 | -4.53108 | -4.57767 |
| mmu-miR-3473 | -0.48039675 | down | -6.23627 | -5.63241 | -5.7513 | -6.5242 | -6.54033 | -6.52246 |
| mmu-miR-100 | -0.47656226 | down | -2.79802 | -2.83936 | -3.07575 | -3.7118 | -3.77404 | -3.66399 |
| mmu-miR-23a | -0.47185868 | down | 0.06949 | 0.399951 | 0.238921 | -0.3285 | -0.40751 | -0.3113 |
| mmu-miR-350 | -0.46377563 | down | -3.24249 | -3.10616 | -3.42331 | -3.9281 | -3.92483 | -4.00759 |
| mmu-miR-378 | -0.4626994 | down | -4.49661 | -4.16328 | -4.45216 | -4.9501 | -4.94995 | -4.95365 |
| mmu-miR-96 | -0.45401978 | down | -2.86051 | -2.42411 | -3.02317 | -3.3132 | -3.25644 | -3.53878 |
| mmu-miR-5097 | -0.45153177 | down | 1.79464 | 1.715739 | 1.613341 | 1.0189 | 0.881386 | 1.128229 |
| mmu-miR-361 | -0.45023346 | down | -4.75466 | -4.52865 | -4.82066 | -5.2872 | -5.26696 | -5.31346 |
| mmu-miR-29b | -0.44518697 | down | -0.88078 | -0.3041 | -0.74589 | -1.1301 | -1.12181 | -1.31648 |
| mmu-miR-5100 | -0.43704224 | down | 2.850779 | 2.970881 | 2.907889 | 2.3776 | 2.192603 | 2.411331 |
| mmu-miR-30a | -0.42991996 | down | -3.70556 | -3.27234 | -3.5089 | -4.0482 | -3.98927 | -4.06626 |
| mmu-miR-677* | -0.41822386 | down | -3.62509 | -3.39673 | -3.63144 | -4.1339 | -4.22792 | -4.07932 |
| mmu-miR-720 | -0.41730762 | down | 3.33506 | 3.371254 | 3.41161 | 2.7832 | 2.612866 | 2.925789 |
| mmu-miR-22 | -0.41554832 | down | -1.96312 | -1.45728 | -1.65286 | -2.1508 | -2.10073 | -2.19975 |
| mmu-miR-21 | -0.41086006 | down | 1.481084 | 1.849856 | 1.745929 | 1.2126 | 1.209112 | 1.213557 |
| mmu-miR-378b | -0.40927243 | down | -4.14159 | -3.86257 | -3.97672 | -4.6089 | -4.40704 | -4.65872 |
| mmu-miR-1949 | -0.40747976 | down | -1.3765 | -1.26215 | -1.43913 | -1.8303 | -2.0713 | -1.80268 |
| mmu-miR-340-5p | -0.40691376 | down | -5.03398 | -4.60373 | -5.06362 | -5.4472 | -5.3503 | -5.63211 |
| mmu-miR-16 | -0.39293414 | down | -0.60521 | -0.36965 | -0.71464 | -1.1631 | -1.16332 | -1.16286 |
| mmu-miR-20b | -0.39115244 | down | -0.83069 | -0.80881 | -0.88233 | -1.486 | -1.57575 | -1.33966 |
| mmu-miR-24 | -0.39044443 | down | 0.026423 | 0.425016 | 0.161827 | -0.3063 | -0.31549 | -0.29715 |
| mmu-miR-10a | -0.37741065 | down | -3.96048 | -3.60563 | -4.03864 | -4.313 | -4.30875 | -4.43039 |
| mmu-miR-107 | -0.3751086 | down | -1.84268 | -1.59423 | -1.96042 | -2.3117 | -2.30943 | -2.32386 |
| mmu-miR-27a | -0.37456578 | down | -0.96837 | -0.64644 | -0.76388 | -1.2974 | -1.29276 | -1.30115 |
| mmu-let-7e | -0.3717792 | down | -2.62896 | -2.50109 | -2.57488 | -3.073 | -3.07547 | -3.02551 |
| mmu-miR-3096b-3p | -0.37173396 | down | -0.98109 | -0.83895 | -0.96084 | -1.5673 | -1.58138 | -1.26821 |
| mmu-let-7a | -0.37043315 | down | 0.455421 | 0.67774 | 0.405229 | -0.0353 | -0.04007 | -0.02808 |
| mmu-miR-34a | -0.37008214 | down | -2.48294 | -2.21333 | -2.67672 | -2.9742 | -2.87935 | -3.04727 |
| mmu-miR-98 | -0.3683777 | down | -3.4191 | -3.24466 | -3.52139 | -3.9507 | -3.94887 | -3.95195 |
| mmu-miR-183 | -0.36583138 | down | -4.80809 | -4.38106 | -4.66183 | -5.0614 | -5.00402 | -5.10694 |
| mmu-miR-301a | -0.36195087 | down | -3.2406 | -2.84483 | -3.29086 | -3.6462 | -3.62778 | -3.66277 |
| mmu-miR-17* | -0.35969257 | down | -4.03034 | -3.95628 | -4.06345 | -4.3865 | -4.66174 | -4.36526 |
| mmu-miR-93 | -0.3594978 | down | -2.10935 | -1.92895 | -2.1685 | -2.5601 | -2.62455 | -2.53543 |
| mmu-miR-709 | -0.35415584 | down | -0.6371 | -0.46713 | -0.62477 | -1.1248 | -1.21538 | -1.0855 |
| mmu-miR-103 | -0.3516518 | down | -1.98668 | -1.65829 | -2.07286 | -2.4184 | -2.38597 | -2.43133 |
| mmu-miR-29a | -0.33210886 | down | -0.79757 | -0.36038 | -0.66236 | -0.9766 | -0.93981 | -1.03149 |
| mmu-miR-3960 | -0.33037663 | down | -3.73113 | -2.90158 | -3.29421 | -3.8291 | -3.82914 | -3.78822 |
| mmu-miR-210 | -0.32627606 | down | -2.75719 | -2.69261 | -3.24385 | -3.3645 | -3.23726 | -4.06801 |
| mmu-miR-674* | -0.32558012 | down | -3.75529 | -3.99746 | -4.09999 | -4.6304 | -4.70126 | -4.3503 |
| mmu-let-7f | -0.32139304 | down | 0.54985 | 0.818326 | 0.52611 | 0.14 | 0.17715 | 0.138887 |
| mmu-miR-151-5p | -0.3160019 | down | -3.52399 | -3.47607 | -3.56893 | -3.9351 | -4.05259 | -3.90536 |
| mmu-miR-140 | -0.30786872 | down | -3.70428 | -3.48079 | -3.73436 | -4.0944 | -4.11343 | -4.08185 |
| mmu-miR-139-5p | -0.30402517 | down | -5.11923 | -4.69998 | -4.81233 | -5.2349 | -5.28545 | -5.23414 |
| mmu-let-7g | -0.29313183 | down | -1.07398 | -0.83546 | -1.17152 | -1.4638 | -1.41224 | -1.51709 |
| mmu-miR-25 | -0.29172063 | down | -2.35146 | -2.32335 | -2.51661 | -2.9305 | -2.95275 | -2.79462 |
| mmu-miR-19b | -0.27977592 | down | 0.444801 | 0.463387 | 0.312301 | -0.0627 | -0.1389 | 0.003119 |
| mmu-let-7b | -0.27154148 | down | -1.15862 | -0.95555 | -1.13094 | -1.4346 | -1.48171 | -1.41769 |
| mmu-miR-99a | -0.26723665 | down | -0.75916 | -0.79586 | -1.15522 | -1.4006 | -1.40319 | -1.38483 |
| mmu-let-7c | -0.2649927 | down | -0.00416 | 0.119061 | -0.12392 | -0.4204 | -0.41359 | -0.42345 |
| mmu-miR-7a | -0.26319218 | down | -2.01089 | -2.16424 | -2.25944 | -2.6181 | -2.69323 | -2.56426 |
| mmu-miR-324-5p | -0.26139832 | down | -4.81258 | -4.30911 | -4.75803 | -4.9679 | -5.00667 | -4.8875 |
| mmu-miR-20a | -0.25832653 | down | -0.0442 | -0.09587 | -0.15542 | -0.4806 | -0.62785 | -0.44346 |
| mmu-miR-322 | -0.256958 | down | -4.85271 | -4.69503 | -5.10108 | -5.1491 | -5.13021 | -5.3852 |
| mmu-miR-652 | -0.24985504 | down | -3.7581 | -3.47425 | -3.84635 | -4.0223 | -4.02762 | -4.01696 |
| mmu-let-7i | -0.24858281 | down | -0.40646 | -0.32271 | -0.49929 | -0.8107 | -0.83622 | -0.76871 |
| mmu-miR-15a | -0.24498689 | down | -1.90555 | -1.65962 | -1.97596 | -2.2003 | -2.15011 | -2.24167 |
| mmu-miR-19a | -0.23176455 | down | -1.35956 | -1.52877 | -1.56555 | -1.9701 | -2.08927 | -1.88537 |
| mmu-let-7d | -0.21324378 | down | -0.7255 | -0.55258 | -0.77661 | -1.0107 | -1.04646 | -0.99593 |
| mmu-miR-106b | -0.17523193 | down | -2.13478 | -1.99776 | -2.29299 | -2.4142 | -2.42556 | -2.41315 |
| mmu-miR-130b | -0.1569829 | down | -2.29987 | -2.18963 | -2.51864 | -2.582 | -2.65438 | -2.57381 |
| mmu-miR-5126 | -0.13445449 | down | -2.396 | -1.58966 | -2.01495 | -2.3257 | -2.29432 | -2.4176 |
| mmu-miR-15b | -0.1316722 | down | -0.5965 | -0.57435 | -0.87909 | -0.9373 | -0.95279 | -0.91359 |
| mmu-miR-92a | -0.12682849 | down | -0.79014 | -0.94531 | -0.8954 | -1.2462 | -1.27008 | -0.98865 |
| mmu-miR-301b | -0.120322704 | down | -6.30345 | -6.01231 | -6.24865 | -6.2928 | -6.28283 | -6.43023 |
| mmu-miR-20a* | -0.10979557 | down | -7.11406 | -6.87392 | -6.74697 | -7.1453 | -7.46948 | -6.51869 |
| mmu-miR-674 | -0.09689951 | down | -6.67746 | -7.03843 | -6.96449 | -7.2763 | -7.42358 | -7.23441 |
| mmu-miR-18a | 0.017598152 | up | -2.03566 | -2.42897 | -2.37904 | -2.5721 | -2.61848 | -2.23328 |
| mmu-miR-2861 | 0.110994816 | up | -5.49667 | -4.63855 | -5.17036 | -5.3534 | -5.29434 | -5.3897 |
| mmu-miR-5128 | 0.76067543 | up | -7.22162 | -6.48954 | -6.74271 | -6.2693 | -6.2787 | -6.23701 |

FC, fold change.
