# Supplementary material for: Rab32 GTPase, as a direct target of miR-30b/c, controls the intracellular survival of Burkholderia pseudomallei by regulating phagosome maturation
Source: PLoS Pathog. 2019 Jun 14;15(6):e1007879. doi: 10.1371/journal.ppat.1007879 (PMC6594657; doi:10.1371/journal.ppat.1007879)
Supplement: S2 Table — (DOCX) [file ppat.1007879.s009.docx]

**S2 Table. Sequences of DNA oligonucleotides and primers used in the paper.**

| **Name** |  | **Sequences** |
| --- | --- | --- |
| **oligonucleotide** |  |  |
| Rab32-3´UTR WT | F | 5´ CTAGTGGGCCCGGCTCTTGTCATACTGCCAAATGCAA ACTAATGTTTACAGCCTTTGAAATAT 3´ |
| Rab32-3´UTR WT | R | 5´ AGCTTATATTTCAAAGGCTGTAAACATTAGTTTGCATT TGGCAGTATGACAAGAGCCGGGCCC 3´ |
| Rab32-3´UTR Mut | F | 5´ CTAGTGGGCCCGGCTCTTGTCATACTGCCAAATGCAA ACTAAACAGGCGAGCCTTTGAAATAT 3´ |
| Rab32-3´UTR Mut | R | 5´ AGCTTATATTTCAAAGGCTCGCCTGTTTAGTTTGCATTT GGCAGTATGACAAGAGCCGGGCCC 3´ |
| **qRT-PCR** |  |  |
| Rab32 | F | 5´ GGATATCGCGGGACAGGAAC 3´ |
| Rab32 | R | 5´ TCGTTTTTCCATTTTAGGACTGC 3´ |
| β-actin | F | 5´GATTACTGCTCTGGCTCCTAGC 3´ |
| β-actin | R | 5´GACTCATCGTACTCCTGCTTGC 3´ |
